# Enrichment of HP1a on Drosophila Chromosome 4 Genes Creates an Alternate Chromatin Structure Critical for Regulation in this Heterochromatic Domain

**DOI:** 10.1371/journal.pgen.1002954

**Published:** 2012-09-20

**Authors:** Nicole C. Riddle, Youngsook L. Jung, Tingting Gu, Artyom A. Alekseyenko, Dalal Asker, Hongxing Gui, Peter V. Kharchenko, Aki Minoda, Annette Plachetka, Yuri B. Schwartz, Michael Y. Tolstorukov, Mitzi I. Kuroda, Vincenzo Pirrotta, Gary H. Karpen, Peter J. Park, Sarah C. R. Elgin

**Affiliations:** 1 Department of Biology, Washington University in St. Louis, St. Louis, Missouri, United States of America; 2 Center for Biomedical Informatics, Harvard Medical School, Boston, Massachusetts, United States of America; 3 Division of Genetics, Department of Medicine, Brigham and Women's Hospital, and Department of Genetics, Harvard Medical School, Boston, Massachusetts, United States of America; 4 Department of Molecular Biology and Biochemistry, Rutgers University, Piscataway, New Jersey, United States of America; 5 Food Science and Technology Department, Faculty of Agriculture, Alexandria University, Alexandria, Egypt; 6 Department of Molecular and Cell Biology, University of California Berkeley, California, United States of America; 7 Department of Genome Dynamics, Lawrence Berkeley National Lab, Berkeley, California, United States of America; 8 Department of Molecular Biology, Umea University, Umea, Sweden; The University of North Carolina at Chapel Hill, United States of America

## Abstract

Chromatin environments differ greatly within a eukaryotic genome, depending on expression state, chromosomal location, and nuclear position. In genomic regions characterized by high repeat content and high gene density, chromatin structure must silence transposable elements but permit expression of embedded genes. We have investigated one such region, chromosome 4 of *Drosophila melanogaster*. Using chromatin-immunoprecipitation followed by microarray (ChIP–chip) analysis, we examined enrichment patterns of 20 histone modifications and 25 chromosomal proteins in S2 and BG3 cells, as well as the changes in several marks resulting from mutations in key proteins. Active genes on chromosome 4 are distinct from those in euchromatin or pericentric heterochromatin: while there is a depletion of silencing marks at the transcription start sites (TSSs), HP1a and H3K9me3, but not H3K9me2, are enriched strongly over gene bodies. Intriguingly, genes on chromosome 4 are less frequently associated with paused polymerase. However, when the chromatin is altered by depleting HP1a or POF, the RNA pol II enrichment patterns of many chromosome 4 genes shift, showing a significant decrease over gene bodies but not at TSSs, accompanied by lower expression of those genes. Chromosome 4 genes have a low incidence of TRL/GAGA factor binding sites and a low T_m_ downstream of the TSS, characteristics that could contribute to a low incidence of RNA polymerase pausing. Our data also indicate that EGG and POF jointly regulate H3K9 methylation and promote HP1a binding over gene bodies, while HP1a targeting and H3K9 methylation are maintained at the repeats by an independent mechanism. The HP1a-enriched, POF-associated chromatin structure over the gene bodies may represent one type of adaptation for genes embedded in repetitive DNA.

## Author Summary

How DNA is packaged into chromatin has profound implications for gene regulation. While certain chromatin conformations are accessible to RNA polymerase and allow expression, other chromatin structures prevent transcription. In many genomes, genes that need to be expressed and repetitive sequences that need to be silenced are interspersed at close intervals. We use *Drosophila melanogaster* chromosome 4 as one example of such a complex domain and ask how the genes on this chromosome are packaged and regulated. While the transcription start sites of active genes on chromosome 4 exhibit the expected pattern of chromatin marks, we see an unusual combination of marks over expressed gene bodies, including enrichment of HP1a and H3K9me3. Deposition of HP1a over the gene bodies is dependent on POF (painting of fourth), while its association with intergenic repeat clusters is accomplished by a different mechanism. In this environment, promoter proximal RNA polymerase pausing is largely absent, despite the fact that genome-wide, approximately 10%–15% of all active genes display pausing. A redistribution of polymerase on chromosome 4 genes, including depletion in the gene body, is observed on HP1a depletion. These findings demonstrate how gene regulation mechanisms can be modulated in specific domains of the genome and illustrate the necessity of examining regulatory pathways within chromatin sub-domains, rather than relying on genome-wide averages or on a limited set of reporter genes.

## Introduction

In eukaryotes, initial gene regulation is achieved through the interaction of transcription factors and the transcriptional machinery with DNA packaged into chromatin. The basic unit of chromatin is the nucleosome, 147 bp of DNA wrapped around a histone octamer [1], [2]. Post-translational modifications of histones, and the presence of core histone variants and additional chromosomal proteins, characterize various chromatin states that determine the accessibility of DNA for transcription. A subset of modifications, such as histone 3 lysine 9 (H3K9) methylation, is associated with gene silencing, while other modifications, such as histone 3 lysine 4 trimethylation (H3K4me3), correlate with gene activity. (For a recent review see [3].) Chromatin states and transcriptional activity are highly regulated to ensure gene activity at the proper developmental time and in the appropriate cell type while maintaining silencing at other, often tightly linked, sequences, including transposable elements (TEs). This need for regulation is particularly evident in genomes similar to the human, where TEs and genes are interspersed, with these repetitious elements found both within and between genes.

In *Drosophila melanogaster*, the small chromosome 4 has an organization that is reminiscent of mammalian genomes. Its 1.2 Mb distal arm hosts approximately 80 genes and has a repeat density of ∼30% [4]. (While the entire chromosome 4 is 4.2 Mb [5], we will refer here to the 1.2 Mb distal arm of chromosome 4 as “chromosome 4”, as the other 3 Mb are composed of highly repetitive sequence for which no genome assembly is available.) Despite a high gene density, similar to that in euchromatic domains in Drosophila, chromosome 4 exhibits hallmarks of heterochromatin. It replicates late [6] and lacks recombination in the laboratory setting, although there is evidence of recombination events over evolutionary time [7], [8]. In most cases reporter transgenes inserted into chromosome 4 are silenced by position effect, resulting in a variegating phenotype [9]–[11]; the entire chromosome is highly enriched for heterochromatic marks such as heterochromatin protein 1a (HP1a) and H3K9 di- and trimethylation [12], [13]. Recent work has demonstrated that chromosome 4 displays distinct chromatin profiles compared to both pericentric heterochromatin and euchromatic regions [14], [15].

How genes are regulated in TE-rich domains with dense heterochromatic marks remains elusive, and Drosophila chromosome 4 provides a system to study the mechanisms involved. Chromosome 4 also provides a potential example of domain-wide regulation with the presence of the painting of fourth (POF) protein, which exclusively binds to the distal 1.2 Mb portion of chromosome 4 [16]. POF binds to gene bodies, and expression of chromosome 4 genes is decreased in its absence [17]. In addition, compensation of gene expression in aneuploid chromosome 4 animals is mediated by POF, supporting its role in gene regulation on this chromosome [18]. Recent work has shown that POF binds nascent RNAs of chromosome 4 genes and that its association with chromosome 4 is dependent on active transcription [19]. Intriguingly, HP1a is enriched in gene bodies with POF, and polytene chromosome analysis has suggested that HP1a and POF are interdependent for deposition on chromosome 4 [17]. Also present on chromosome 4 are two H3K9 histone methyltransferases (HMTs), EGG – a SETDB1 class enzyme – and SU(VAR)3-9 [13], [20]–[22]. SU(VAR)3-9 has been suggested to be enzymatically inactive on chromosome 4 (its main function appears to be in pericentric heterochromatin), while EGG appears responsible for maintaining the bulk of the H3K9me2 and H3K9me3 in this domain [20]–[22]. Co-immunoprecipitation experiments indicate that POF can interact with EGG [22], suggesting that regulation of gene expression on chromosome 4 involves EGG, POF, and HP1a.

To further explore how genes function in a heterochromatin-like milieu in general, and on Drosophila chromosome 4 specifically, we examined the enrichment profiles of 20 histone modifications and 25 chromosomal proteins, drawing on new as well as previously published datasets profiled by the model organism Encyclopedia of DNA Elements (modENCODE) *Drosophila* group [23]. In addition, we mapped H3K9me2/3, H3K36me3, HP1a, POF, and RNA polymerase II (RNA pol II) by chromatin immunoprecipitation-microarray (ChIP-chip) technology in mutant larvae lacking HP1a, POF, or EGG. Our results indicate that chromosome 4 genes are governed by a unique regulatory system characterized by a lack of RNA polymerase pausing, which may be a consequence of the presence of HP1a. We find that efficient POF recruitment is dependent on EGG, but not HP1a. Our results argue that HP1a is recruited to chromosome 4 by two mechanisms: the majority of HP1a (associated with genes) is dependent on POF, while a smaller fraction (associated with TE-rich regions) is POF-independent. We suggest a model where EGG, POF, and HP1a bind to active genes on chromosome 4 and together positively regulate their expression.

## Results

### Mapping of additional chromatin components confirms that chromosome 4 is a distinct heterochromatic domain, rich in transcribed genes

Earlier studies of chromosome 4 using cytological approaches established the enrichment of HP1a and noted a banded pattern, suggesting interspersed domains of low HP1a density that might favor gene expression. However, while low-resolution mapping with an *hsp70-white* reporter transgene indicated a few permissive domains (allowing full expression, red eye), the bulk of the insertions, including 12 within genes, resulted in a variegating phenotype, indicating heterochromatin packaging [9]–[11].

**Figure 1:**
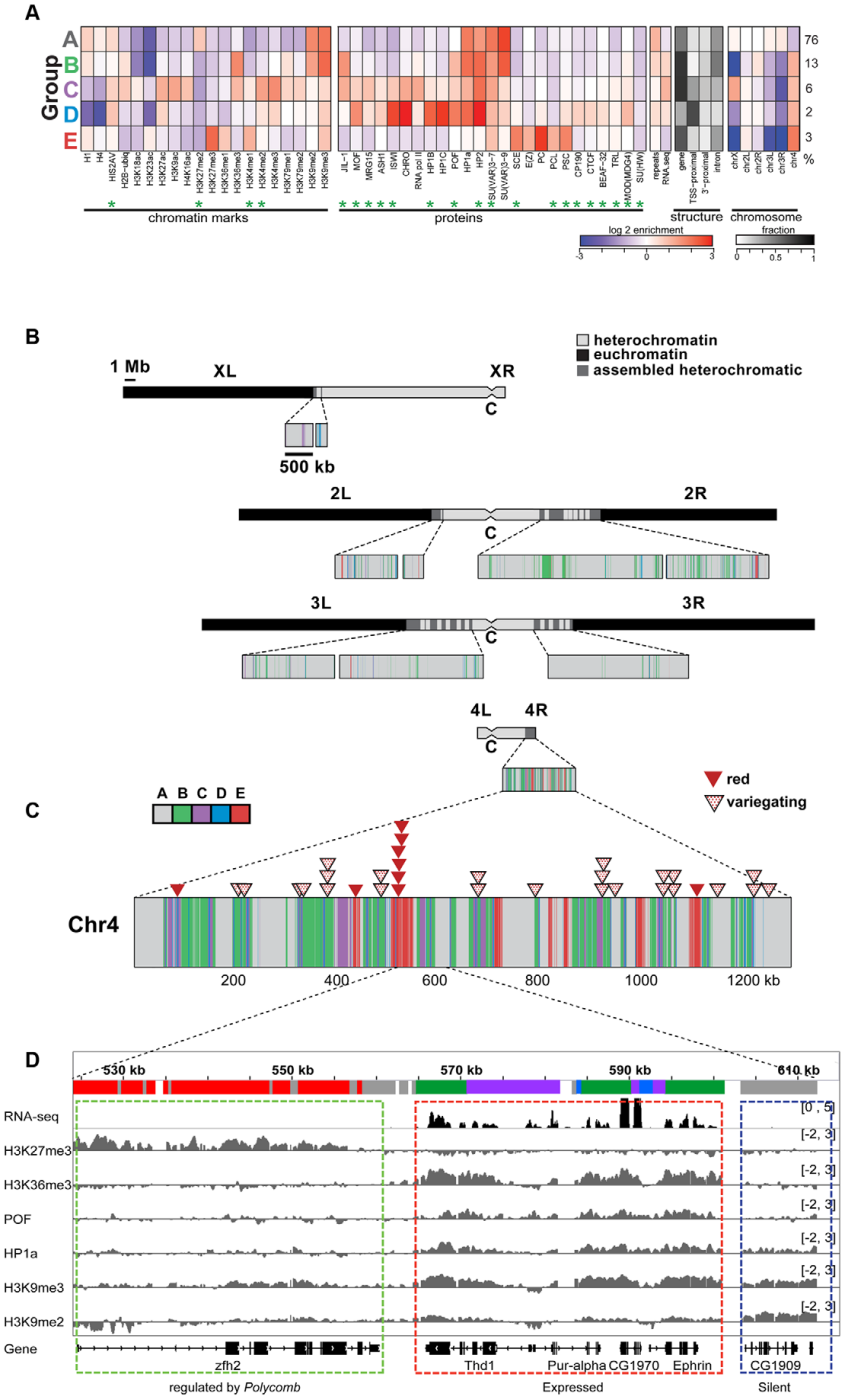
The chromatin composition of *D. melanogaster* chromosome 4 shows distinct patterns of enrichment. A. Enrichment levels for all histone marks/chromosomal proteins (green asterisks indicate newly reported marks) are shown for the five main combinatorial chromatin states within heterochromatin as defined in Riddle et al [15]. Panel 1- histone marks; panel 2- chromosomal proteins; panel 3- repeat enrichment and expression status; panel 4- the fraction of chromatin within each state associated with various structural features of genes; panel 5- enrichment/depletion of states for each chromosome arm, with the numbers to the right reflecting the percentage of the heterochromatin assigned to each state. Chromatin source: BG3 cells. B. Karyotype view of the assembled heterochromatic domains defined by the five combinatorial chromatin states in A. The distal 1.2 Mb region of chromosome 4 exhibits a higher density of transcriptionally active genes (states B, C, D) and polycomb-dominated domains (state E). Chromatin source: BG3 cells. C. *hsp70-white* transgenes leading to a red eye phenotype are preferentially found in state E. Triangles mapped onto the expanded chromosome 4 indicate the insertion sites for *hsp70-white* transgenes exhibiting a red (red triangle) or variegating (dotted triangle) eye phenotype [9]. D. Browser shot illustrating the relationship between histone marks and chromosomal proteins on chromosome 4. The silent gene *CG1909* shows correlations typical of pericentric heterochromatin (dashed blue box; enriched H3K9me2, H3K9me3, HP1a; state A), while the expressed gene *Ephrin* shows the patterns typical for chromosome 4 active genes (dashed red box; upstream promoter regions, depleted for H1 and H4, state D; regions immediately downstream from TSSs, enriched for H3K4me2/3 and depleted of H3K9me2/3, state C; and regions across the body of the gene, enriched for H3K36me3, H3K9me3, HP1a and POF, state B). Silent gene *zfh* shows the chromatin pattern typical for genes under the regulation of Polycomb system, enriched in H3K27me3 and depleted of HP1a.

More recently, we used high-resolution genome-wide enrichment profiles of 16 histone marks and two proteins to identify and map predominant combinatorial chromatin states within heterochromatin [15]. Here, we expand this analysis to include four additional histone marks and 18 additional chromosomal proteins, whose enrichment in the original five predominant combinatorial chromatin states of heterochromatin is shown in Figure 1A. [Throughout this article, we will define pericentric heterochromatin by enrichment in H3K9me2 as described in [15].] Several of the new proteins are enriched in heterochromatin states preferentially found on chromosome 4 (Figure 1A, states B–E). For example, chromosome 4 contains higher levels of POF, JIL-1, MOD(MDG4), HIS2AV (Figure 1A, states B–D), and some *Polycomb*-associated proteins (Figure 1A, state E) compared to pericentric heterochromatin in BG3 cells (Figure 1B). (For S2 cell data see Figure S1.) Compared to the pericentric heterochromatin of other chromosomes, chromosome 4 contains less chromatin in state A, which represents the classical heterochromatin enriched for H3K9me2, H3K9me3, HP1a, HP2, SU(VAR)3-7, and SU(VAR)3-9 (Figure 1A, panel 5). Mapping these chromatin states across the karyotype (Figure 1B and 1C) and at higher resolution across chromosome 4 (Figure 1D) suggests a distinct domain with a higher gene (exon) density in the distal portion of chromosome 4.

Interestingly, we do not see any evidence for euchromatic domains, as defined by depletion of H3K9me2/H3K9me3/HP1a and association with activation marks. Such domains had been suggested by the full expression of an *hsp70*-*white* transgene reporter (red eye phenotype) at certain sites [10], [24]. Rather, we observe a strong correlation between these permissive sites and regions regulated by the *Polycomb* (PcG) system (Figure 1C and Figure S2). PcG regulated genes can be associated with a number of alternative chromatin states, including a repressive state (enriched for Polycomb [PC]), an active state (enriched for ASH1 and TRX), and a void state (lacking PC, ASH1, and TRX) [25]. The insertion sites of all red-eyed reporters correspond to four regions that in some cell types lack H3K9 methylation and HP1a, but contain H3K27me3 and PC. In contrast, none of the 24 *hsp70-white* reporter lines with a variegating eye phenotype are found in regions associated with PC in the cells and tissues examined to date (Figure S2B). While it is unknown which PcG chromatin state is present at the insertion sites of red-eyed reporter lines in the developing eye, our results confirm that HP1a and PC occupy separate domains on chromosome 4 and suggest that the domains regulated by the *Polycomb* system are transcription-permissive for *hsp70-white* reporters in the critical cell type. In contrast, the bulk of the genes on chromosome 4 are associated with HP1a, a well-established mark of heterochromatin, correlated with silencing. To explore how these genes function, we looked further at the distribution of chromosomal proteins associated with these genes.

### Active genes on chromosome 4 are characterized by a distinct combination of POF, H3K36me3, HP1a, and H3K9me2/3

**Figure 2:**
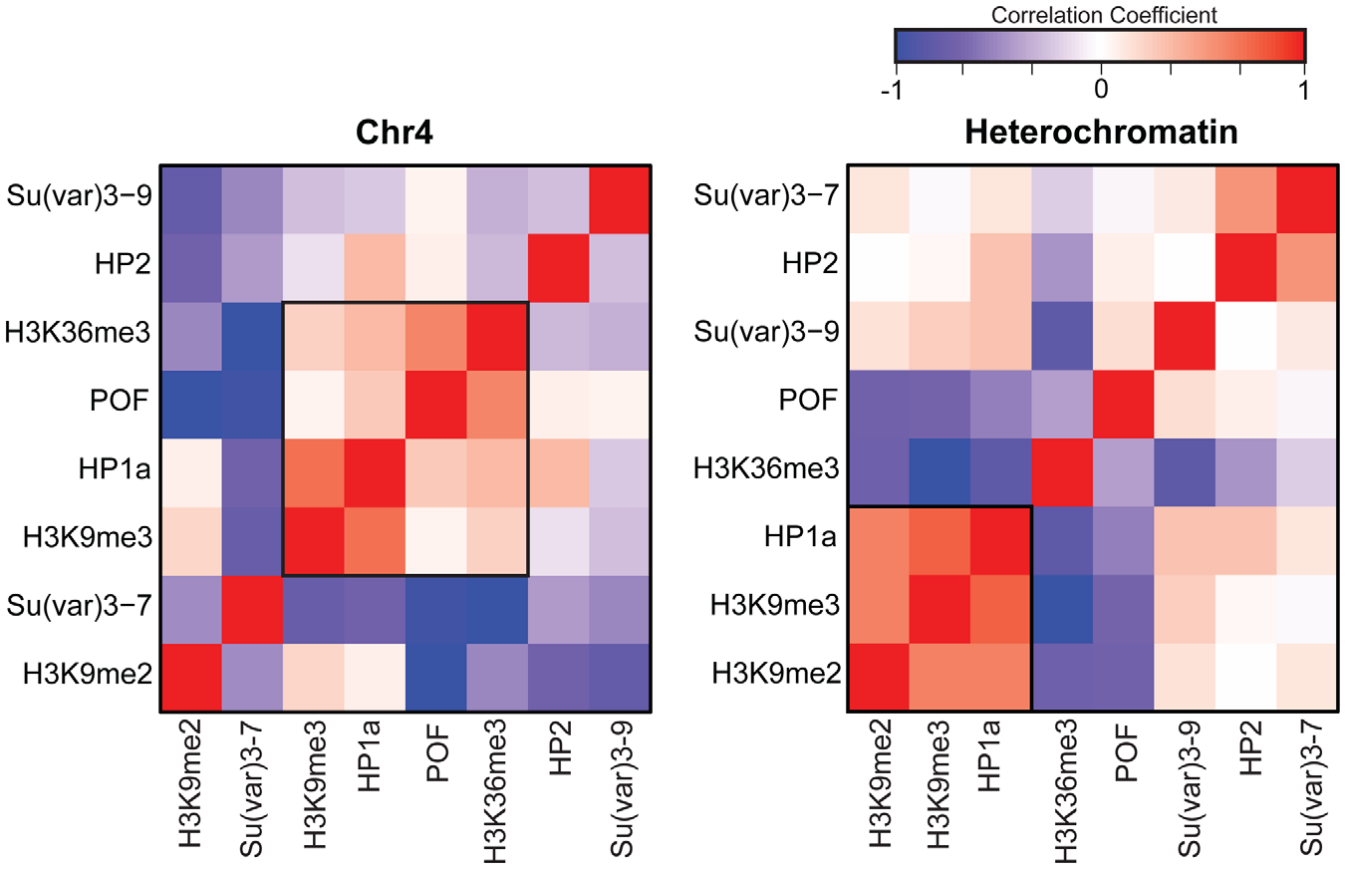
The relationship between marks of classical heterochromatin and gene expression are altered on chromosome 4. The strength of correlation between marks is illustrated in this diagram by the color intensity (red - positive correlation; blue - negative correlation). In pericentric heterochromatin, the black outline demarcates the strong correlation structure observed between H3K9me2, H3K9me3, and HP1a (right). This strong correlation is not present on chromosome 4; HP1a and H3K9me3 instead are positively correlated with H3K36me3, a mark of elongation, and the chromosome 4-specific protein POF (left).

Previous work by us and by others has indicated that HP1a correlates well with H3K9me2 and H3K9me3 in pericentric heterochromatin [14], [15]. However, H3K9me2 and H3K9me3 have distinct distributions on chromosome 4 (Figure 1A, compare states A–E), leading us to re-examine the correlation of these marks as well as a few others in chromosome 4 and pericentric heterochromatin. While pericentric heterochromatin maintains the expected association among silencing marks, we find that HP1a and H3K9me3 correlate positively with active marks POF and H3K36me3 on chromosome 4 (Figure 2). Other marks associated with silencing (H3K9me2, SU(VAR)3-9, SU(VAR)3-7, and HP2) show little or no correlation on the chromosome 4.

**Figure 3:**
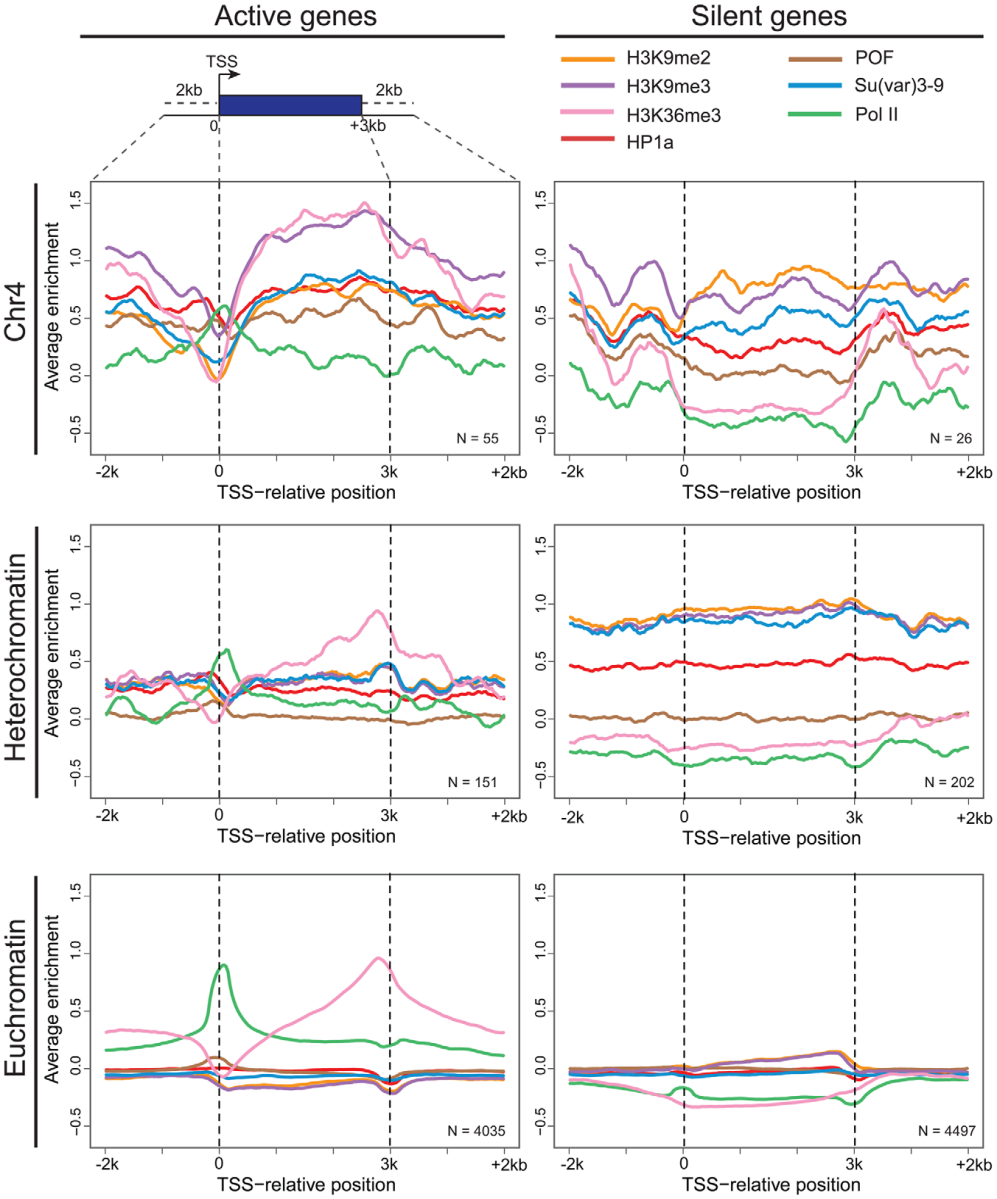
Metagene analysis shows a unique distribution of chromosomal proteins and histone marks on chromosome 4 genes. Enrichment (averaged smoothed M-values, Y-axis) for select chromosomal proteins and histone marks is plotted for a 3 kb scaled metagene (bp, X-axis). The enrichment is examined in three genomic domains: Chromosome 4 (top); pericentric heterochromatin (middle); and euchromatin (bottom) for active genes (left column) and repressed genes (right). Active genes on chromosome 4 have distinctive signatures of HP1a, POF, and H3K9me3 with highest enrichment levels across gene bodies. The number of genes included is demonstrated at the right corner for each figure. Data from BG3 cells.

Given that chromosome 4 is distinguished from pericentric heterochromatin by its higher gene density, we hypothesized that the change in the correlation patterns is related to genes specifically. Thus, we examined the “metagene” profiles for active and silent genes on chromosome 4, within pericentric heterochromatin, and in euchromatin. (Active and silent genes were defined by RNA-seq data, as described in Materials and Methods.) Indeed, the correlated histone modifications and proteins noted above map together only on chromosome 4, enriched over the body of active genes, in contrast to what is observed at other active loci (Figure 3, results from BG3 cells; see Figure S3 for data from S2 cells). This difference is not due to the relatively small number of genes present on chromosome 4, but is also seen when the same number of genes are compared for chromosome 4, heterochromatin, and euchromatin (metagenes in Figure S4 and S5, heatmaps in Figure S6). H3K9me2 is the only mark on chromosome 4 preferentially associated with repressed gene bodies. The high levels of POF and HP1a associated with transcribed genes on chromosome 4 confirm prior findings by Johannson and colleagues [17]. The enrichment of H3K9me3 in these regions of active transcription is unexpected and suggests a unique mechanism regulating H3K9 methylation on chromosome 4.

### Chromosome 4 genes rarely display RNA polymerase pausing

**Figure 4:**
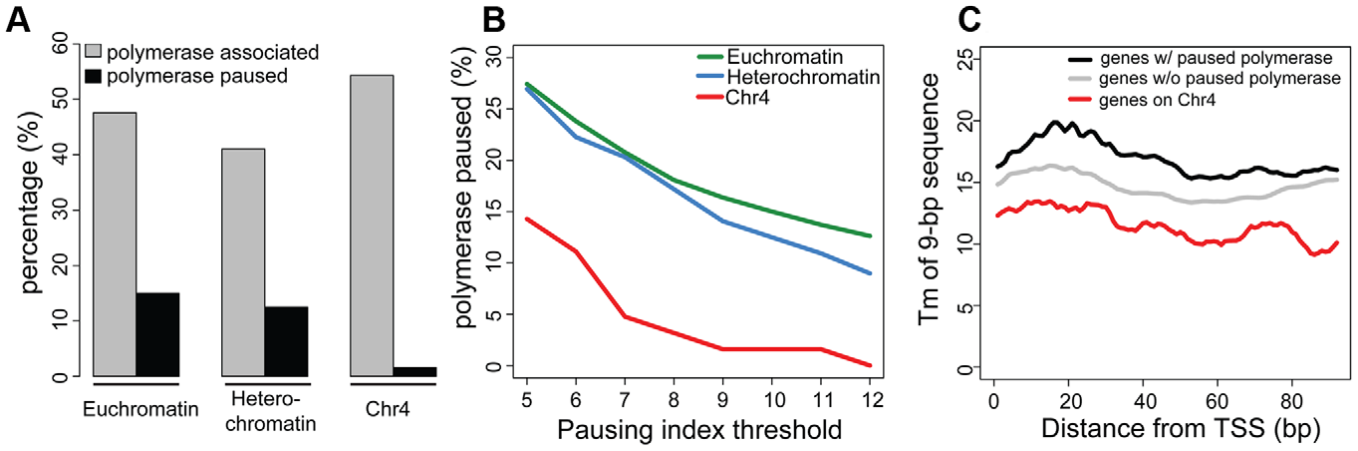
Chromosome 4 has a very low incidence of polymerase pausing identified by GRO-seq data. A. The bar graph shows the percentage of transcripts associated with RNA polymerase (grey) and the percentage of those RNA polymerase-associated genes exhibiting significant pausing (black) for euchromatin, pericentric heterochromatin, and chromosome 4. A pausing index (PI) threshold value of 10 was used. B. The low frequency of polymerase pausing observed for chromosome 4 is independent of the PI selected. C. The average T_m_ of 9-mers downstream of the TSS for chromosome 4 genes tends to be lower than that of other genes. T_m_ (Y-axis) of 9-mers in the 100 bp downstream of the TSS (bp, X-axis) is compared for genes on chromosome 4 (red) to genes classified as either paused (black) or not-paused (grey) by GRO-seq analysis. Data from S2 cells [23].

As previously reported, silencing marks are depleted at the TSSs [15]. Figure 3 compares the chromatin composition at the TSS and the gene body for chromosome 4 genes. The distinctive enrichment patterns observed for TSSs and gene bodies suggested a possible role for this chromatin structure in regulation at the TSS. Given the anticipated difficulty in transcribing through a region with HP1a and H3K9me3, we considered changes in polymerase dynamics, such as pausing, to be likely affected. For a significant number of active genes, RNA pol II initiates transcription but pauses after 25–50 nt, remaining there until pausing is relieved. We investigated polymerase association with genes and polymerase pausing on chromosome 4 using global run-on followed by sequencing (GRO-seq) with data from S2 cells produced by Larschan and colleagues [26]. First, we compared the association of polymerase with genes in euchromatin, pericentric heterochromatin, and chromosome 4. RNA-seq data derived from steady state mRNA revealed that, while pericentric heterochromatin has a lower gene density, the fraction of active genes is roughly the same between heterochromatin (pericentric heterochromatin and chromosome 4) and euchromatin (54% vs. 52% in S2 cells). GRO-seq data confirmed this assessment, indicating that 47.6% of euchromatic genes were being actively transcribed in S2 cells, compared to 40.4% of those in heterochromatin. On chromosome 4, 54.3% of the genes were associated with GRO-seq signal, a fraction slightly higher but not significantly different from that of euchromatin (p = 0.147; Figure 4A). However, the GRO-seq signal on chromosome 4 and within euchromatin was higher than that in pericentric heterochromatin (p<0.01; Figure 4A).

Next, we assessed polymerase pausing using a pausing index (PI) that measures the level of polymerase at the 5′ end of the gene compared to that over the gene body [27]. Specifically, we used the ratio of GRO-seq read density in the first 500 bp of the gene and the read density of the first 25% of the remaining length of the gene (for details, see [26]). With a stringent threshold for pausing, the results indicate that in pericentric heterochromatin and in euchromatin, the fraction of genes associated with a paused polymerase is similar, 12.5% and 15.0%, respectively (Figure 4A). In contrast, only 1.6% of the RNA polymerase-associated chromosome 4 genes exhibit such pausing, a significantly smaller percentage than what was observed in either euchromatin or pericentric heterochromatin (p<0.0005; Figure S7). While the absolute number of paused genes varies depending on the threshold, the difference in pausing frequency between chromosome 4 and the other genome domains using this analytical definition was observed over a wide range of PI thresholds (Figure 4B). This finding is consistent with recent results from Johannsen and colleagues using the same GRO-seq dataset [19]. We also validated this result using ChIP-chip data from S2 and BG3 cells with an alternative definition of pausing (see Materials and Methods; Table S3). The overlap in the genes identified as exhibiting pausing by these two methods is ∼50%, significantly more than the random expectation (p<1×10^−16^, Figure S8). These data demonstrate that paused polymerase (no matter how defined) is not uniformly distributed across genomic domains, and further establish the unique properties of chromosome 4. Whether the observations on chromosome 4 reflect a difference in the protein machinery identified with “classical” RNA pol II pausing (i.e. dependent on NELF and DSIF) or with some other aspect of RNA pol II regulation (e.g. elongation) remains to be explored. Given that our observations are based on experimental approaches widely used to study pausing, and for convenience, we will refer to this particular distribution of RNA pol II as ‘pausing’ in the text below.

### Sequence characteristics of chromosome 4 are congruent with its low level of paused polymerase

Previous work has shown that chromosome 4 is distinct from other domains in a number of sequence-associated features [4], [28]. As various gene features (e.g. sequence composition [29], gene expression levels [27], and gene ontology [30], [31]) have also been associated with the use of polymerase pausing, we considered whether these features of chromosome 4 might be correlated with the observed lack of polymerase pausing. “Developmental control” genes (gene ontology), which preferentially exhibit pausing [30], [31], occur on chromosome 4 at the same frequency as in other genomic domains (Table S4), and the average copy number for chromosome 4 segments in S2 cells is similar to that of the other autosomes [32]. The low fraction of genes displaying pausing is also not due to proximity to the centromere: the percentage of genes displaying pausing within the first 1.2 Mb of contiguously assembled sequence on chromosome arms 2L, 2R, and 3L is 13.6% in S2 cells. Genes on chromosome 4 are larger than those in euchromatin and heterochromatin (median: 8,001 bp vs. 1,907 bp vs. 1,844 bp; Wilcoxon test: p<0.0001, Figure S9A and S9D), and the average number of exons for genes on chromosome 4 is higher than for genes in euchromatin and pericentric heterochromatin (median: 6 vs. 3 vs. 2; p<0.0001; Figure S9B and S9E). However, larger, more exon-rich genes tend to have higher PI values (Figure S6E), indicating that this feature does not contribute to the lack of pausing on chromosome 4. Genes on chromosome 4 can be biased towards higher expression levels (e.g. in third instar larvae: p<0.05; Figure S9C; no higher expression in S2 cells), but this bias is cell type-specific, while the lack of pausing is observed in all cell types examined. Thus, these sequence features alone cannot account for the low incidence of polymerase pausing observed on chromosome 4.

Previous reports have identified several sequences that are preferentially associated with paused polymerase, including GAGA factor (TRL) binding motifs, the Inr motif, as well as the so-called “pause button” (PB) sequence KCGRWCG [29]. We find that the fraction of promoters with a PB or Inr motif is similar in euchromatin and on chromosome 4 (Table S5), and thus unlikely to contribute to the differences we observe in pausing incidence. In contrast, the fraction of promoters containing TRL motifs (or their inversions) differs significantly between euchromatin and chromosome 4. While TRL binding sites are observed in 24% of euchromatic promoters, we find TRL binding sites in only 11% of the promoters on chromosome 4 (Table S5; p<2.69e-3; inverted TRL binding site: 25% vs. 13%, p<4.88e-3). The underrepresentation of TRL binding sites on chromosome 4 is reflected also in the significantly lower number of TRL-bound sites detected in TRL ChIP-chip data. In euchromatin, 33% of promoters are bound by TRL, while on chromosome 4 only 17% show TRL binding in S2 cells (p<1.52e-3). Thus, the paucity of TRL binding sites on chromosome 4 might contribute to the low occurrence of polymerase pausing, but cannot explain it entirely.

Recently, it has been reported that genes exhibiting polymerase pausing have a distinct T_m_ (melting temperature) peak for 9-mers approximately 25–30 bp downstream of the TSS [33]. Therefore, we examined T_m_ values of 9-mers in the TSS-proximal 100 bp of each *D. melanogaster* mRNA by a sliding window analysis. Interestingly, the T_m_ values for TSS-associated sequences on chromosome 4 are lower than those on the other chromosomes for both pausing and non-pausing genes (11.50 degrees vs. 15.03–15.84 degrees; see Table S6) over the entire 100 bp interval (see Figure 4C). Thus, chromosome 4 genes show a different sequence organization at their 5′ end, which may contribute to the low incidence of pausing.

### Lack of HP1a alters RNA pol II distribution and decreases gene expression levels on chromosome 4

**Figure 5:**
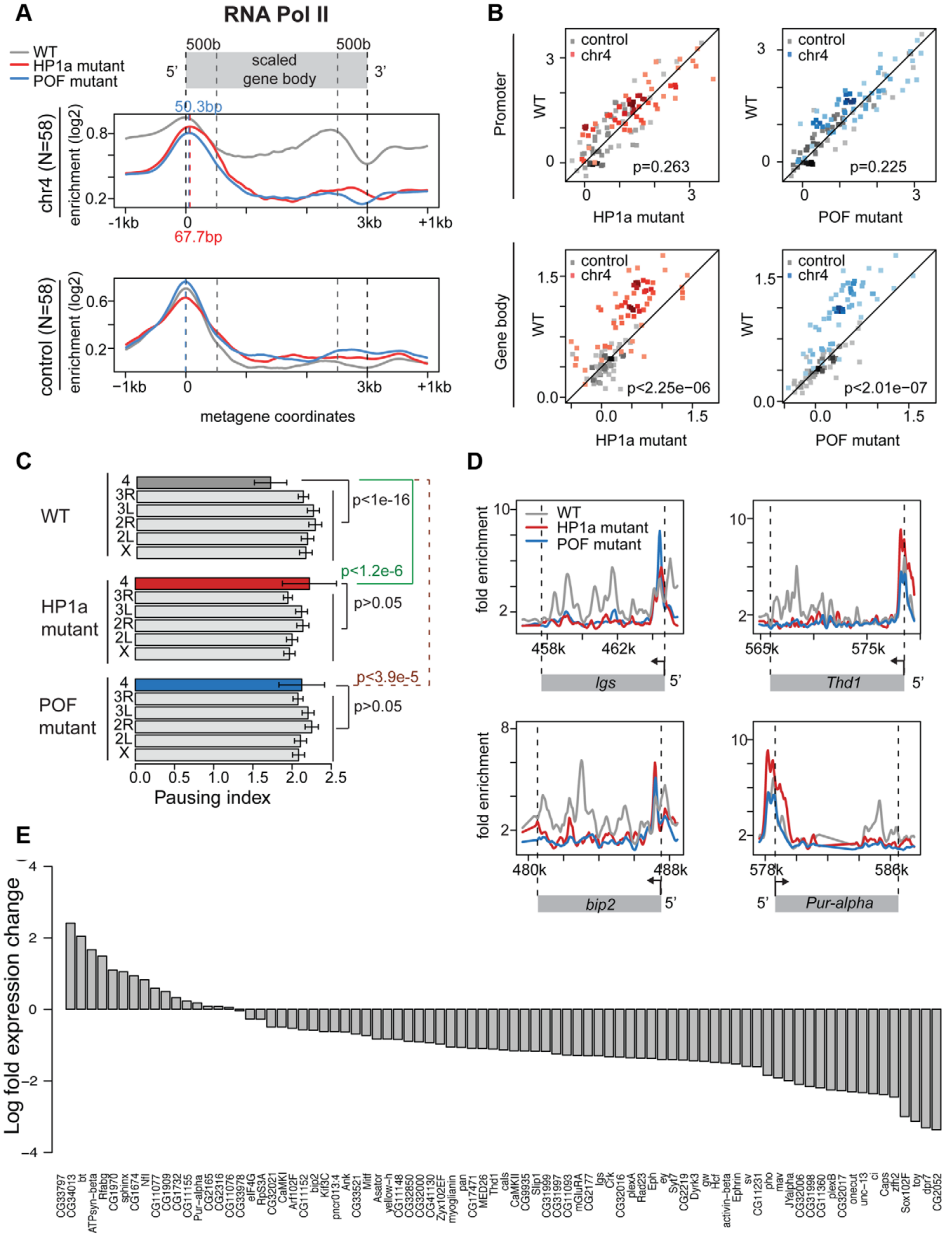
Lack of HP1a or POF shifts the enrichment pattern of RNA pol II on chromosome 4. A. RNA pol II distribution of expressed genes on chromosome 4, comparing wildtype (WT) to HP1a (red) and POF (blue) mutants. Chromosome 4 genes are compared to genes with similar expression levels randomly chosen from the euchromatin on chromosome arm 3R (N = 58 for both). Note the dramatic loss of RNA pol II enrichment in the gene body of the mutants for chromosome 4 genes, but not for the control euchromatic genes. The TSS-proximal RNA pol II peak also changes in the mutants compared to wildtype, moving approximately 68 bp and 50 bp downstream, respectively. B. Scatter plot showing enrichment of RNA pol II at the promoter region (+/−500 bp of TSS, top panel) and gene body (bottom panel). In both HP1a and POF mutants, the RNA pol II enrichment decreases significantly only in the gene body. C. Pausing index (PI) changes specifically on chromosome 4 in HP1a and POF mutants. In both mutants, PI for chromosome 4 genes increases to a range similar to the other chromosomes. D. Examples of the shift of RNA pol II in four chromosome 4 gene regions in the mutants. In *Pur-alpha*, no probes were available from 581 kb–584 kb. E. Fold changes of expression level (FPKM) of chromosome 4 genes in the HP1a mutant compared to the wildtype. Data from third instar larvae.

Given the low frequency of paused polymerase on chromosome 4, but not in pericentric regions of heterochromatin, we tested the hypothesis that chromosome 4's distinct chromatin composition is responsible for this difference. First, we disrupted the typical chromatin structure, using third instar larvae lacking HP1a, trans-heterozygous for *Su(var)205^04^* and *Su(var)205^05^*. These trans-heterozygotes do not produce zygotic HP1a and survive to the third larval instar by utilizing maternally loaded HP1a protein and/or mRNA. By the third instar, little detectable HP1a protein remains, and in ChIP-chip experiments, >95% of peaks observed in wildtype are absent in the mutants (Figure S10A). RNA pol II enrichment on chromosome 4 in *Su(var)205* mutants is reduced in the gene bodies, leading to an increase in PI, as RNA pol II now is relatively more concentrated at the TSS compared to the wildtype distribution (Figure 5A, compare red [HP1a −/−] to grey [+/+]). For RNA pol II ChIP-chip data, the PI is defined as the ratio between the maximum enrichment value around the TSS (+/−300 bp) and the medium enrichment values over the gene body (600 bp downstream of the TSS to the end of the gene) [31]. Analyzing the changes in RNA pol II distribution separately for the promoter region and the gene body, we find that only the changes in the gene body are significant (Figure 5B; p<2.25e-6). The shift to TSS-biased enrichment is illustrated for several genes in Figure 5D. While the level of RNA pol II enrichment at the promoter does not change in the HP1a mutant, the location of the peak shifts by approximately 68 bp into the gene body, a position suggestive of a paused polymerase. In total, 54 of 73 genes larger than 600 bp on chromosome 4 show an increase in PI (calculated as in [31] for ChIP-chip data, Figure S11), which is significantly different from what we observe for the remainder of the genome (Figure 5C, p<1.2e-6). Thus, the shift in RNA pol II distribution is specific to chromosome 4 genes and does not occur at euchromatic genes in general.

Next, we examined the effect of HP1a depletion on gene expression using RNA-seq data. On chromosome 4, there is a significant overall decrease in the expression level (Figure S12D; p<2.26e-6, paired Wilcoxon test; p = 0.205 on chromosome 3R, a control euchromatic region). Specifically, 67 of 84 genes (∼80%) exhibit decreased expression upon HP1a depletion (Figure 5D) in contrast to ∼60% genome-wide. Expression is significantly decreased for *plexB*, *ci*, *CG31998*, *dpr7*, *Lin29*, *zfh2*, *onecut*, *mav*, *CG11360*, *Sox102F*, *unc-13*, *toy*, *CG32017*, *pho*, and *Caps* (FDR = 0.01), while significantly increased expression is observed for *CG1970*, *bt*, *ATPsyn-beta*, and *Rfabg* (FDR = 0.01). Among the genes showing decreased expression in the HP1a mutant, a group of ∼10 genes also loses H3K36me3 signal relative to wildtype. In addition, genes on chromosome 4 with decreased expression levels tend to show depletion of RNA pol II in *Su(var)205 [HP1a]* mutants (Figure S11C). The loss in expression after HP1a depletion indicates that most chromosome 4 genes are behaving as “heterochromatic genes” by this criterion [34].

Using an RNA-immunoprecipitation technique, Piacentini and colleagues have identified a set of euchromatic genes regulated by HP1a in S2 cells [35]. We compared the response of this gene set to HP1a depletion in HP1a mutant larvae to the response of chromosome 4 genes (Figure S12). While chromosome 4 genes show a strong reduction of RNA pol II over the gene bodies in the absence of HP1a (p<7.52e-7), the putative euchromatic HP1a-regulated genes show a slight increase in RNA pol II enrichment (p = 0.02). In addition, the euchromatic gene set did not show a reduction in gene expression in these larvae [*Su(var)205^04^/Su(var)205^05^*]. Our exceptional findings for chromosome 4 genes demonstrate a unique role for HP1a in this domain, affecting RNA pol II distribution as well as overall gene expression. The data indicate that the specific, high enrichment of HP1a over actively transcribed gene bodies on chromosome 4 positively regulates gene expression, and reduces 5′ accumulation of RNA polymerase at these genes, possibly by promoting elongation and/or by interfering with pausing.

### POF mutants also show an altered RNA pol II distribution on chromosome 4

We speculated that disrupting chromosome 4's unique chromatin structure by removing POF would also affect the RNA pol II distribution. Thus, we investigated the relationship between POF and polymerase pausing using homozygous *pof^D119^* third instar larvae. *pof^D119^* is a null mutant that lacks the first and part of the second exon of the POF coding sequence [36]. 79% of POF enrichment seen in wildtype is absent in *pof* mutants (Figure S10B). The gene body POF signal is absent in the mutant, while the remaining signal is TSS-associated and most likely represents cross-reactivity. As observed for HP1a mutants, we see a shift from a broader distribution of RNA pol II in the wildtype to a TSS-biased enrichment in the *pof* mutant (Figure 5A and 5B), with a significant decrease of RNA pol II enrichment over the gene body (p<2.01e-7). Thus, most genes on chromosome 4 (63 of 73) show an increase in PI, similar to what we observed in mutants lacking HP1a (Figure S13). The PI changes on chromosome 4 (relative to wildtype) are significant (p<3.9e-5), but those for the remainder of the genome are not (Figure 5C). Also similar to HP1a mutants, this shift in RNA pol II distribution, specifically the reduction of RNA pol II over the gene body, is unique to chromosome 4.

To develop a better understanding of the genes undergoing a shift in RNA pol II distribution in the HP1a and POF mutants, we examined their association with various chromosomal proteins in wildtype (Figure S14). We find no exceptional enrichment for any of the additional chromosomal proteins we examined. Correlating the change in PI observed for the chromosome 4 genes in the HP1a and POF mutants with protein enrichment levels yields only low correlation values, with the highest value being r = 0.39 for RPD3 (Figure S14B). Together, our data from the HP1a and POF mutants suggest that chromosome 4 genes specifically respond to the disruption of their unique chromatin environment. That environment is dominated by high levels of HP1a over actively transcribing gene bodies, along with enrichment of POF – conditions that surprisingly result in RNA pol II enrichment over the gene bodies and low pausing indices.

### HP1a recruitment to chromosome 4 occurs by two mechanisms, one POF-dependent, one POF-independent

**Figure 6:**
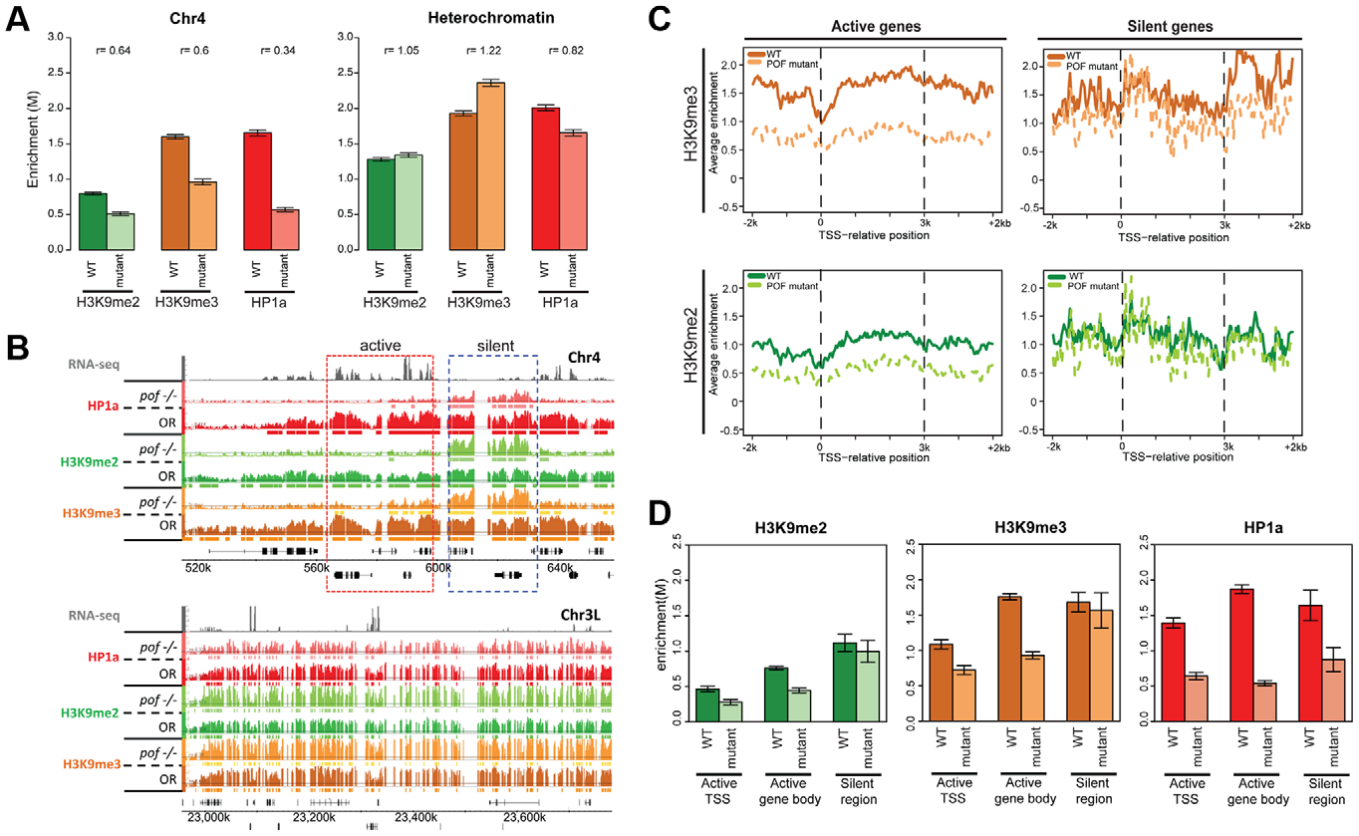
Lack of POF leads to large-scale changes in HP1a and H3K9me2/3 and demonstrates that HP1a on chromosome 4 consists of POF-dependent and -independent pools. A. Mutations in POF alter H3K9me2, H3K9me3 and HP1a enrichment on chromosome 4. Enrichment levels (M-values) are shown for H3K9me2, H3K9me3, and HP1a on chromosome 4 (left) and in pericentric heterochromatin (right) in wildtype (dark color) and *pof^D119^* homozygous mutant (light color) third instar larvae. Error bars: Standard error of the mean (SEM). B. Browser shots illustrating the loss of HP1a on chromosome 4 (top) in *pof^D119^* homozygous mutant third instar larvae and the retention of high levels of HP1a in pericentric heterochromatin (bottom panel). The M-value scale (Y-axis) is identical for wildtype and mutant but differs between marks (0–3 for H3K9me2 and H3K9me3; 0–4 for HP1a). C. Metagene plots showing H3K9me3 and H3K9me2 levels are reduced mainly over active genes on chromosome 4 in the mutant. Genes on chromosome 4 were divided into transcriptionally active (left column) and transcriptionally silent (right) based on RNA-seq data. D. The changes in H3K9me2, H3K9me3, and HP1a enrichment induced by the *pof* mutation correlate with gene features on chromosome 4. Changes in H3K9me2/me3 and HP1a enrichment (Y-axis: smoothed M-values) are examined separately for TSSs of actively transcribed genes, gene bodies of active genes, and silent regions on chromosome 4. Error bars: SEM.

It is likely that loss of POF or HP1a has a profound impact on the overall chromatin composition of chromosome 4, as suggested by the altered polymerase dynamics. To test this hypothesis, we investigated the relationship between EGG, POF, HP1a, H3K9me2, and H3K9me3 in several mutants at the third instar larval stage. ChIP-chip analysis of *pof^D119^* chromatin reveals that over 90% of the HP1a enrichment observed on chromosome 4 in wildtype is abolished, and HP1a is now enriched in scattered peaks rather than the very broad domains observed in wildtype (Figure 6B, upper panel); these peaks are absent in profiles from HP1a mutants. Pericentric heterochromatin of chromosomes X, 2, and 3 (not normally associated with POF) retains strong enrichment for HP1a (Figure 6B, lower panel). Furthermore, we find that the strong HP1a enrichment over active gene bodies seen on wildtype chromosome 4 is lacking in *pof* mutant larvae (Figure 6B and 6D). Interestingly, the HP1a peaks remaining in *pof* mutants are located near repeats, with a median distance to repeats of 38 bp, significantly shorter than the 132 bp expected under a random distribution derived from chromosome 4 (Figure S15A, p<0.001). (Note that our assay is restricted to uniquely mapping sequences and does not score identical repeats. Due to the incomplete genome assembly, it is formally possible that some of the sequences attributed to chromosome 4 also exist in the unassembled portions of the Drosophila genome.) This finding indicates that there are two mechanisms recruiting HP1a to chromosome 4: the majority of the HP1a recruitment is POF-dependent, but a significant amount of HP1a recruitment is targeted to repeat clusters, and this recruitment is independent of POF.

### H3K9me2 and H3K9me3 levels are reduced on the transcribed genes of chromosome 4 in *pof^D119^* mutants

ChIP-chip data also reveal abnormal H3K9 methylation patterns in *pof* mutant larvae on chromosome 4 (Figure 6 and Table S7). In wildtype, H3K9me2 and H3K9me3 are enriched along chromosome 4, with H3K9me3 enriched most strongly over transcribed gene bodies, mimicking POF and HP1a. In *pof* mutants, the extent of the H3K9me2 and H3K9me3 enriched domains on chromosome 4 is reduced chromosome-wide by 40% and 59%, respectively, regardless of wildtype POF enrichment levels in these domains (POF+/POF−; Table S7). The enrichment levels of H3K9me2 and H3K9me3 on chromosome 4 are decreased by 36% and 40%, respectively (Figure 6A), whereas in pericentric heterochromatin they are slightly increased in the *pof* mutant (Figure 6A). H3K9me2 and H3K9me3 enrichment levels are significantly decreased in actively transcribed regions (37% and 49% reductions), with little change observed in silent regions (p>0.05, Figure 6C and 6D). These results indicate that POF has a positive effect on H3K9me2/3 enrichment only on chromosome 4 and specifically in transcribed domains. The regions of H3K9me2/me3 enrichment that remain in the *pof* mutant correlate well with the remaining HP1a enrichment regions, with 82.8% of the HP1a enriched sequences found embedded in H3K9me2 regions, and 98% of the HP1a enriched regions overlapping with H3K9me3 regions. This positive correlation resembles that observed in pericentric heterochromatin; one now sees higher levels of HP1a, H3K9me2, and H3K9me3 over the intergenic and silent gene regions. The overlap suggests that the remaining H3K9 methylation might serve as a “seed” to recruit the residual HP1a observed in repeat-rich regions of chromosome 4, but that the recruitment of HP1a to the body of active genes requires POF. Alternatively, it is possible that HP1a is directly recruited to repetitive sequences, and can then recruit the necessary enzymes for generating the H3K9 methylation in these domains.

### POF deposition is independent of HP1a

**Figure 7:**
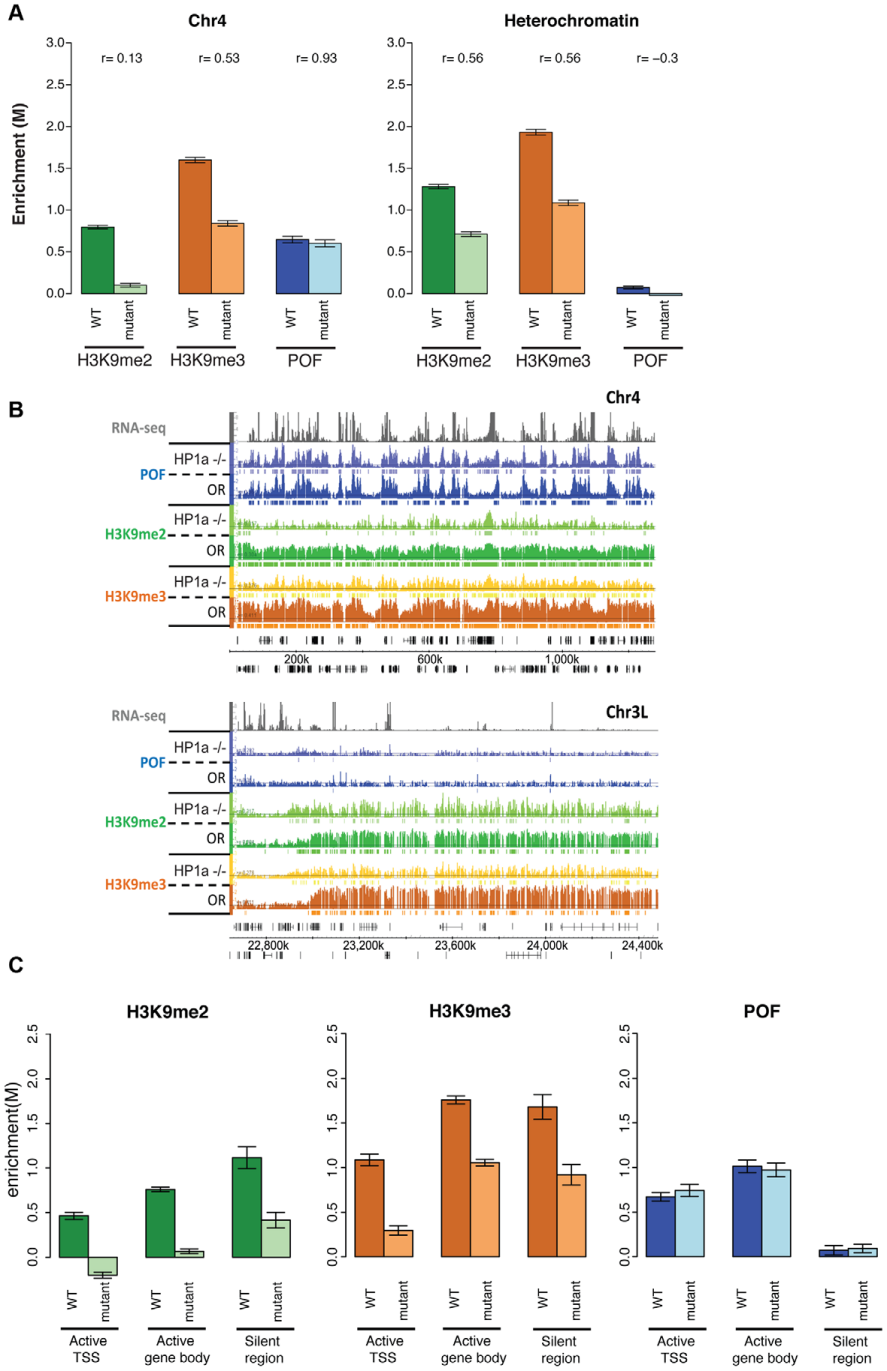
Lack of HP1a does not lead to a loss of POF from chromosome 4. A. H3K9me2 and H3K9me3 levels decrease in HP1a mutants, while POF enrichment is not reduced. The smoothed M-value (Y-axis) is shown for pericentric heterochromatin (right) and chromosome 4 (left) comparing wildtype (dark color) and trans-heterozygous *Su(var)205^04^/Su(var)205^05^* mutants. Error bars: SEM. B. Browser shot illustrating the retention of POF enrichment on chromosome 4 in HP1a mutants (top panel) and depletion of H3K9me2 and H3K9me3 both in chromosome 4 (top) and pericentric heterochromatin (bottom). The M-value scale (Y-axis) is identical for wildtype and mutant ranging from 0 to 3. C. Changes in H3K9me2, H3K9me3, and HP1a enrichment at TSSs of actively transcribed genes, over gene bodies of active genes, and in silent regions on chromosome 4 in HP1a mutant. Error bars: SEM.

Polytene chromosome analysis had suggested that HP1a and POF enrichment on chromosome 4 are interdependent [22], [36]. In order to verify this at a higher resolution, we carried out POF ChIP-chip analysis in HP1a mutants. As expected, ∼94% of the HP1a enrichment in wildtype is absent in the trans-heterozygous mutant third instar larvae (*Su(var)205^04^/Su(var)205^05^*), both in pericentric heterochromatin and on chromosome 4. However, the POF distribution and its enrichment levels on chromosome 4 are unaffected in this mutant strain (p>0.05; Figure 7 and Table S7). This finding implies that POF recruitment to chromosome 4 is largely independent of HP1a. Alternatively, HP1a could be involved in an initial recruitment in the early embryo (when heterochromatin is formed), but be unessential for maintenance of POF association.

### H3K9me2/3 distributions on chromosome 4 depend both on HP1a-dependent and independent mechanisms

The significantly altered H3K9 methylation on chromosome 4 in the *pof* mutants suggests the possibility of a similar effect in mutants lacking HP1a, leading us to investigate H3K9 methylation levels on chromosome 4 and in pericentric heterochromatin in HP1a mutants. We find that both H3K9me2 and H3K9me3 are significantly decreased in pericentric heterochromatin (p<0.001, Figure 7A). Significant depletion of H3K9me2 (to 11.1% of wildtype) and H3K9me3 (to 33.3% of wildtype) is seen on chromosome 4 as well (Figure 7A and Table S7). With the exception of the first (centromere-proximal) 70 kb of assembled chromosome 4 sequence (discussed below), the regions of H3K9me3 enrichment that remain in the HP1a mutant are correlated with POF binding sites (69.1% retained in POF-positive regions while 30.9% retained in POF-negative regions, Table S7). In contrast, H3K9me2 is lost at similar rates in POF-positive and –negative portions of chromosome 4. Overall, it appears that HP1a is required in pericentric heterochromatin and chromosome 4 for wildtype levels of H3K9 methylation, but that a low level of both methyl marks is able to persist in the absence of HP1a. We suggest the possibility that the HP1a-dependent H3K9 methylation is mediated by the HP1a-interacting H3K9 HMT SU(VAR)3-9 [13], while the residual H3K9 methylation observed in the mutant is mediated by a different HMT, such as EGG or G9a.

### EGG is required for recruitment and/or maintenance of POF and HP1a at the majority of binding sites on chromosome 4

**Figure 8:**
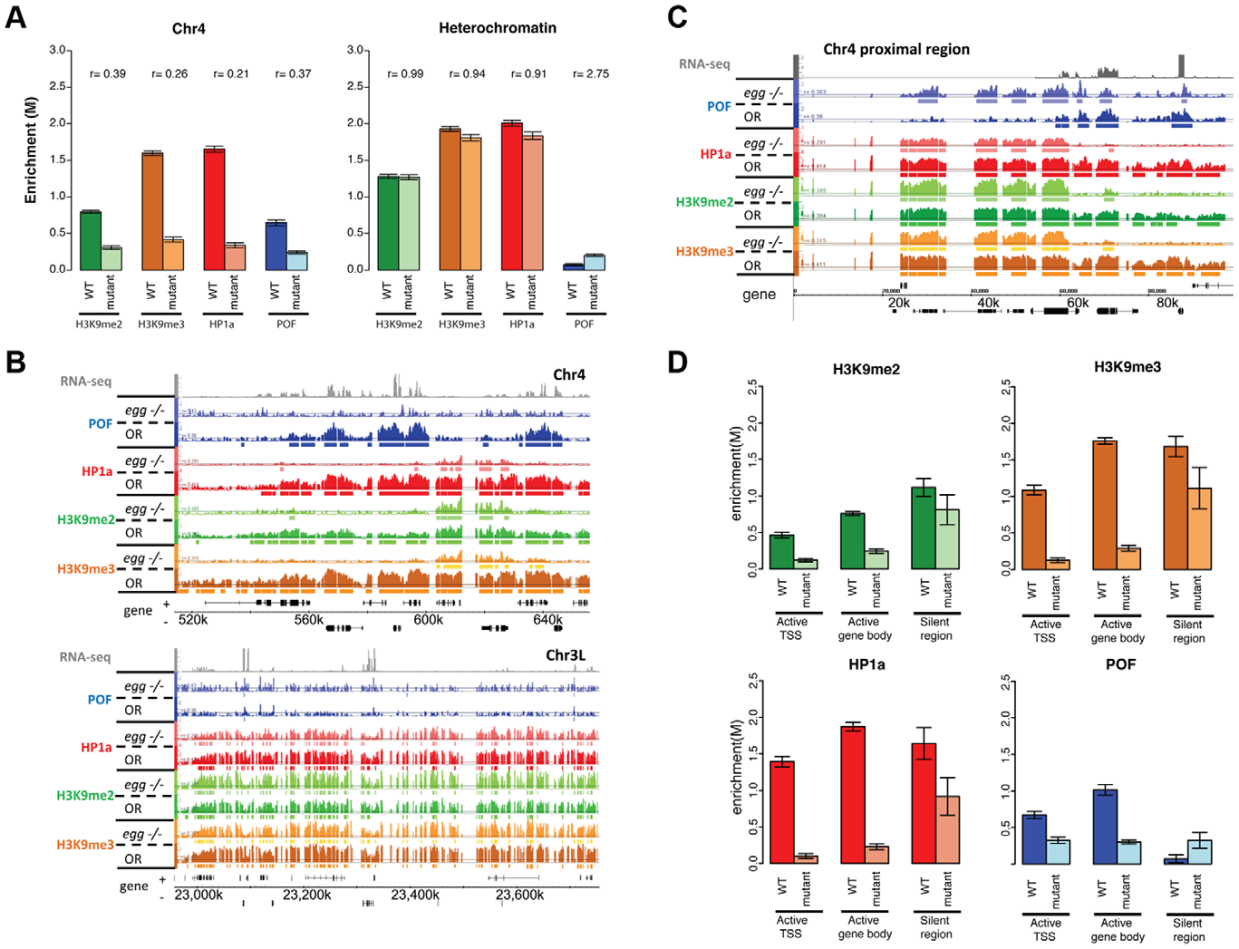
Lack of EGG leads to large-scale changes in POF, HP1a, and H3K9 methylation specifically on chromosome 4. A. Depletion of EGG alters H3K9me2/3, HP1a, and POF enrichment. Scaled enrichment is shown on chromosome 4 (left) and in pericentric heterochromatin (right) comparing wildtype (dark color) and *egg 10.1-1a* homozygous mutant (light color). Error bars: SEM. B. Browser shots showing the reduction of POF, HP1a, and H3K9me2/3 on chromosome 4 (top panel) and the relatively small change in pericentric heterochromatin observed in *egg* mutants. The M-value scale (Y-axis) is identical for wildtype and mutant ranging from 0 to 3. C. The 70 kb proximal region on chromosome 4 shows minimal changes in POF, HP1a, and H3K9me2/3 levels in *egg* mutants, distinct from the alterations in the remainder of the chromosome illustrated in B. The M-value scale (Y-axis) is identical for wildtype and mutant ranging from 0 to 3. D. Changes in H3K9me2/me3, HP1a, and POF enrichment (Y-axis: smoothed M-values) are examined separately for TSS of actively transcribed genes, their gene bodies, and silent regions on chromosome 4. Error bars: SEM. Data from third instar larvae.

The altered H3K9 methylation in mutants lacking POF or HP1a led us to consider the involvement of the H3K9 HMTs in generating the distinct chromatin structure of chromosome 4. EGG is the Drosophila SETDB1 class H3K9 histone methyltransferase, and it has been reported to be a major H3K9 methylation-producing methyltransferase on chromosome 4 based on immunohistochemistry and position effect variegation experiments [20]–[22], [37]. Examining chromatin from homozygous *egg^10.1-1a^* third instar larvae ([21]; null mutants, derived from a heterozygous stock carrying a GFP balancer), we find a number of significant changes in enrichment profiles compared to wildtype, primarily on chromosome 4 (Figure 8A and 8B). Overall levels of POF were significantly depleted (decreased by 63%, Figure 8A), with only 18% of binding sites remaining on chromosome 4 (Table S7). Similarly, the HP1a-enriched regions were reduced by 83.2% and the level of enrichment of HP1a was decreased by 79% (Table S7, Figure 8A). These findings are consistent with the depletion of POF and HP1a seen on *egg* mutant polytene chromosomes [22]. Interestingly, some strong HP1a binding sites remain, which suggests that recruitment of HP1a to these sites is independent of EGG (Figure 8B). 63.9% of the HP1a peaks remaining in *egg* mutants coincide with HP1a peaks retained in the *pof* mutant (Table S7). HP1a peaks retained in *egg* mutants (and *pof* mutants) are within TE-rich regions (medium distance to a TE is 19 bp compared to the 135 bp of random expectation, p<0.001, Figure S15B). Thus, the enrichment profiles from *egg* mutants suggest that EGG is required for the majority of the recruitment and/or maintenance of POF and HP1a at actively transcribed genes, but not at some repeats.

### Loss of EGG protein alters the distribution of H3K9me2 and H3K9me3 on chromosome 4

We also investigated the effects of reduced EGG levels on H3K9 methylation. In *egg^10.1-1a^* mutants, we observed a significant reduction of H3K9me2 and H3K9me3 on chromosome 4 (decreased by 61% and 84%, respectively in Figure 8A, Table S7). While the overall H3K9me2/me3 level on transcribed genes of chromosome 4 dropped significantly (Figure 8B), there were several residual enriched areas, where H3K9me2/me3 was maintained despite the absence of EGG (22.7%/19.4% enriched regions remaining respectively, Table S7; Figure 8B, top panel; and Figure 8D). The remaining H3K9me2 and H3K9me3 enrichment is similar to that observed in the *pof* mutant (Figure 6). This finding implies that these residual H3K9me2/me3 enriched domains are produced by an H3K9 HMT other than EGG. However, whether this activity is restricted to the mutant condition or is present in the wildtype as well is currently unclear and will require further experiments. It is interesting to note that the residual H3K9me2 enriched regions coincide with regions of residual HP1a binding in this mutant. As HP1a is known to bind to H3K9me2/H3K9me3, this finding suggests that the residual H3K9me2/me3 is capable of recruiting HP1a in the absence of EGG and POF. Conversely, the presence of HP1a could recruit an HMT such as SU(VAR)3-9, a known HP1a binding protein, to the region, resulting in H3K9 methylation.

### The ∼70 kb closest to the centromere in the assembled sequence of chromosome 4 is a pericentric-heterochromatin-like domain where HP1a, H3K9me2, and H3K9me3 deposition are independent of POF and EGG

In several of our analyses the most centromere-proximal portion of the assembled chromosome 4 sequences shows a response to the depletion of the various proteins that is clearly distinct from that of the remainder of the chromosome. For example, in chromatin from third instar larvae lacking POF (*pof^D119^*), HP1a, H3K9me2 and H3K9me3 are maintained at a level and density similar to wildtype in the ∼70 kb of assembled sequence adjacent to the centromere (Figure S16). A similar effect is seen in EGG mutant larvae - HP1a and H3K9 methylation are reduced along most of chromosome 4, but maintained in this same ∼70 kb region (Figure 8C). Overall, this domain behaves similarly to the pericentric heterochromatin regions of chromosomes X, 2, and 3, where mutations in *pof* and *egg* do not affect the enrichment of HP1a and H3K9 methylation. This finding suggests that the proximal ∼70 kb of chromosome 4 can be considered to be pericentric heterochromatin, with chromatin characteristics distinct from those of the remainder of distal chromosome 4. Our data support the conclusion that enrichment of HP1a, H3K9me2, and H3K9me3 in pericentric heterochromatin (including the basal portion of chromosome 4) is established by a different mechanism than the enrichment of these same marks over active gene bodies on chromosome 4. Inter-genic clusters of repeats on chromosome 4 are likely to be assembled into heterochromatin by the same mechanism as that operating in pericentric regions.

## Discussion

The genome-wide enrichment profiles of 20 histone modifications and 25 chromosomal proteins demonstrate the distinct nature of chromatin on Drosophila chromosome 4. As anticipated based on the behavior of transgene reporters [10], [11], [24], we found that chromosome 4 sequences are almost ubiquitously packaged with marks commonly associated with heterochromatin, H3K9me2, H3K9me3, HP1a, and HP2 (Figure 1). The TSSs of active genes are depleted for these marks (Figure 3). Surprisingly, “permissive” domains, which allow full expression of reporter genes, were found not to resemble euchromatin, but to show evidence of *Polycomb* regulation (associated with H3K27me3 and PC in some cell types) (Figure 1). The association with *Polycomb* marks is cell-type specific; thus, some genes on chromosome 4 appear to be able to switch between the two main silencing systems in what appears to be a developmentally regulated process. We do not know the state of the *Polycomb* regulated domains in the cells of the eye imaginal disc, where *white* reporter expression is required to result in a red eye phenotype. It is possible that in these cells the *Polycomb* regulated domains are associated with its activating antagonist, *trithorax*, and its partners. However, packaging in the PC state, which appears to exclude HP1a and H3K9me2/3 in this situation, is sufficient to allow DNase1 hypersensitive site (DH site) formation at the genes in these domains in BG3 cells, while such sites are not evident when the same genes are packaged with HP1a and H3K9me2/3 in S2 cells (modENCODE data tracks; www.modENCODE.org). Given that loss of DH site formation has been observed for the variegating reporter [38], a domain that permits DH site formation may be sufficient for reporter expression.

Due to the unusual chromatin environment, chromosome 4 genes experience a unique regulatory system and display decreased polymerase pausing (Figure 4). Mutant analysis indicates that the RNA pol II distribution pattern is dependent on HP1a (Figure 5). In mutants lacking HP1a or POF, enrichment of RNA pol II decreases in the gene bodies, leading to an increase in PI due to the now strongly TSS-biased RNA pol II distribution (Figure 5). This shift in RNA pol II seen in *pof* mutants is potentially an indirect effect due to the inability to recruit HP1a to active gene bodies in the absence of POF. Alternatively, both HP1a and POF together might be required for the wildtype RNA pol II distribution – and the decrease of polymerase pausing - normally seen on chromosome 4. How HP1a, and possibly POF, influence polymerase distribution is still unknown. This influence might occur at various steps of RNA pol II regulation, either by interfering with the establishment, maintenance, or resolution of the paused polymerase, or by promoting elongation. For example, the Positive Transcription Elongation Factor b (P-TEFb) and PAF1C act by promoting elongation [39]. On the other hand, Min and colleagues found that in mouse embryonic stem cells, “bivalent” genes associated with PRC1 and PRC2 display low levels of polymerase pausing, possibly due to their chromatin structure [40]. POF's influence could be mediated by its RNA recognition motif [16] and its ability to interact with RNA transcripts [19], leading to a positive effect on gene expression [19], [36]. Our data, however, indicate that POF alone is insufficient to determine the RNA pol II distribution on chromosome 4 genes, and that HP1a is vital for their regulation. Thus, further work is needed to elucidate the exact mechanism of interaction between POF, HP1a, and the polymerase.

Another protein to consider in the regulation of chromatin structure and RNA polymerase distribution on chromosome 4 genes is JIL-1, which is enriched on chromosome 4 (Figure 1). JIL-1 is an H3S10 kinase; it limits heterochromatin extent, as in its absence, HP1a and H3K9me2 spread to new genomic regions [41]–[44]. Depletion of JIL-1 overall has little effect on gene expression [45], with the major effect being on the X chromosome, with approximately 10% of the genes affected, based on our analysis. In contrast, ∼5% of the chromosome 4 genes are affected, less than the percentage of X chromosome genes but slightly more than seen in the remainder of the genome. As in HP1a and POF mutants, the expression of the affected genes decreases. However, given the small number of genes affected by JIL-1 depletion, the impacts of HP1a/POF depletion are unlikely to be dependent on JIL-1. This interpretation is supported by the genetic interaction analysis of JIL-1 and HP1a, which indicates that their mutations counteract each other's effects, and that the spread of H3K9me2 triggered by Jil-1 mutations is not dependent on HP1a [46].

While HP1a is best known for its role in heterochromatin formation and silencing, several reports have also linked HP1a to regulation of transcriptional activity of both heterochromatic and some euchromatic genes [47]–[49]. Heterochromatic genes *light* and *rolled* are reported to be dependent on a heterochromatic environment, and specifically on HP1a, for optimal expression [34], [50], and we find that the majority of the chromosome 4 genes show a similar dependence (Figure 5D). The distribution of H3K9me2/me3 at several active heterochromatic genes shows depletion at the TSS [51], as reported here for chromosome 4 genes. However, it has recently been reported that two chromosome 4 genes, *CAPS* and *Dyrk3*, lose DNase accessibility at the 5′ DH site in the absence of HP1a [52]. Thus, while absence of HP1a and other silencing marks from the TSS is associated with gene expression in heterochromatic and chromosome 4 genes, the presence of HP1a in the domain as a whole appears to be required for DH site formation at these genes. In contrast, HP1a domains are prohibitive for DH site formation at the TSSs of eukaryotic reporter genes inserted into these regions [38].

In euchromatin, we have found HP1a associated with a number of TSSs, a finding that is supported by the detection of small amounts of HP1a in chromosome arms of polytene chromosomes [12]. Others have identified HP1a as a positive regulator of more than 100 genes, associating with the transcript and apparently facilitating elongation [35]. HP1a has been reported to interact with dKDM4, an H3K36 demethylase, [53], whose yeast homologs promote transcript elongation [54]. Thus, there are precedents for an “activating” role for HP1a, and an interaction with dKDM4 provides an attractive model for how HP1a might influence RNA pol II processivity and pausing. However, what remains to be determined is why polymerase pausing would be affected specifically on chromosome 4 rather than also affecting genes in pericentric heterochromatin. We note that while the overall pericentromeric domains are strongly enriched for HP1a, one does not see the increase over the gene body observed for the chromosome 4, and hence these genes do not exhibit the same contrast between TSS and gene body observed for chromosome 4 genes (Figure 3). POF may play a role in enhancing HP1a presence at active genes on chromosome 4.

The chromatin structure analysis we present from mutants lacking POF, HP1a, and EGG is mostly in agreement with previously published results based on polytene chromosome analysis. On chromosome 4, lack of POF induces loss of HP1a [36], H3K9me2 [22], and H3K9me3 (our study). However, our higher resolution analysis reveals a pool of HP1a, H3K9me2, and H3K9me3 associated with repeated sequences on chromosome 4 that is independent of POF. Also in contrast to prior findings [36], our results indicate that POF is maintained on chromosome 4 independent of HP1a, as mutants lacking HP1a still show a normal POF enrichment pattern. Note that HP1a depletion was accomplished here by a heteroallelic cross; thus HP1a was present during the initial assembly of heterochromatin.

It has been postulated that POF is recruited to chromosome 4 from a site close to the centromere of the chromosome, based on translocation studies [55]. However, the affinity of POF for transcribed genes leads to an enrichment pattern that changes from cell type to cell type, arguing against a simple recruitment and spreading model (comparison of modENCODE data from Bg3 and S2 cells). Our analysis of mutants (resulting in depletion) of *Su(var)205* (HP1a), *egg*, and *pof* products instead suggests a model where there is a simultaneous requirement for EGG and POF, which together create conditions to recruit HP1a to active gene bodies on chromosome 4, presumably utilizing H3K9 methylation by EGG. EGG and POF are reported to physically interact with each other *in vivo* [22], providing a mechanism for this process. How the complex is targeted to chromosome 4 active genes remains to be established.

An interesting aspect of our study is that on chromosome 4, the association between HP1a, H3K9me2, and H3K9me3 is substantially different from what is observed in pericentric heterochromatin (Figure 2). The loss of the strong correlation between H3K9me2 and H3K9me3 on chromosome 4 is likely due to differences in H3K9 HMTs. While little is known about G9a, both EGG and SU(VAR)3-9 have been examined in our study and by others [20]–[22], [56], [57]. Both EGG and SU(VAR)3-9 are found on chromosome 4, but the predominant H3K9 methylation signal depends on EGG [20]–[22], [37]. Our analysis suggests that H3K9me2 and H3K9me3 enrichment on chromosome 4 reflects both HP1a-dependent and HP1a-independent mechanisms. H3K9me2 and H3K9me3 enrichment on chromosome 4 reflects an EGG-dependent mechanism to modify the histone over the body of the genes, and an EGG-independent mechanism to modify the histone associated with repeat sequences (Figure 8). Presumably the latter reaction is carried out by SU(VAR)3-9. We suggest that this activity of SU(VAR)3-9 was missed in the polytene chromosome studies of *Su(var)3-9* mutants [13], as EGG appears to be responsible for ∼80% of the H3K9me2/me3 signal on chromosome 4 in our analysis. Currently, it is unclear how the HMT activities on chromosome 4 are coordinated. In HeLa cells, several H3K9 HMTs interact with each other [58], thus providing potential mechanisms for coordination. However, how the enzymes on Drosophila chromosome 4 produce the H3K9me2 and H3K9me3 enrichment pattern as well as the active gene-specific increase in H3K9me3 remains to be discovered.

**Figure 9:**
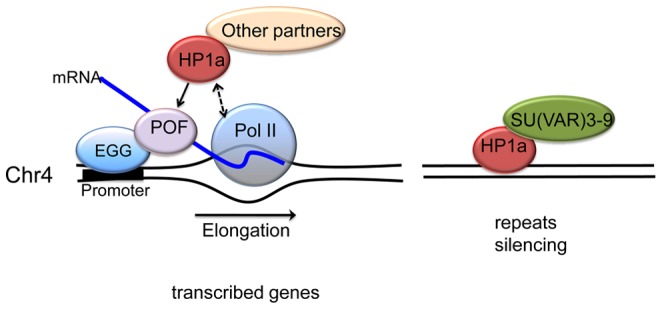
A model illustrating the two mechanisms proposed for HP1a assembly on chromosome 4. In active transcribed regions, POF and EGG are recruited, which leads to high levels of HP1a across the gene body (left). Details are unknown; POF is not reported to interact directly with HP1a, so other protein partners may be involved, in addition to HP1a binding to H3K9me2/3. The chromatin complex containing POF, HP1a and other partners has a general positive effect on RNA pol II – possibly by affecting transcription elongation - specifically on chromosome 4. In silent, repeat-rich regions, which lack POF enrichment, H3K9me marks deposited by a second histone methyltransferase (presumably SU(VAR)3-9) lead to a POF-independent assembly of HP1a–containing chromatin (right). Note that neither EGG, POF, nor SU(VAR)3-9 are known to interact directly with DNA; the binding described occurs in a chromatin context.

The available data suggest the following model for the assembly of chromatin on chromosome 4 and regulation of the genes in this domain (Figure 9). Two mechanisms recruit HP1a to chromosome 4, one dependent on POF and EGG, the other independent of these components. POF is required for the recruitment of HP1a and H3K9 methylation in gene bodies of actively transcribed genes, and EGG appears to be required for significant recruitment or stabilization of POF. POF in turn interacts with the nascent transcript, positively affecting transcript output. Neither POF nor EGG is required for the recruitment of HP1a and the presence of H3K9me2/me3 at repeat clusters (and silent genes) on the chromosome 4. These findings suggest that the same general mechanisms that result in heterochromatic packaging of repetitious, TE-derived DNA in pericentric heterochromatin are at work here as well. Studies in plants and some fungi suggest that small RNAs play a role in targeting heterochromatin formation, and there is growing evidence for such a mechanism establishing heterochromatin patterns in the germline and early embryo of Drosophila [59], [60]. However, direct targeting of one of the heterochromatin components by other means (such as direct DNA recognition) remains a possibility. The analysis above clearly shows that chromosome 4 is a mosaic of HP1a-associated domains, with each of the two modes of assembly detected here potentially impacting gene expression.

## Materials and Methods

Datasets used are listed in Table S1 (cell lines) and Table S2 (mutants). Flybase version 5.12 was used for all analyses. Additional details on materials and methods can be found at www.modENCODE.org.

### Fly stocks and culture conditions

Fly stocks were maintained on standard cornmeal media at 25°C with 70% humidity [61]. Mutant third instar larvae lacking HP1a were recovered from a cross of flies carrying the *Su(var)205^04^* allele [62] over a GFP balancer to flies carrying the *Su(var)205^05^* allele [62] over a GFP balancer by selecting for lack of GFP. Mutant third instar larvae lacking POF were recovered from a homozygous stock of the *pof^D119^* allele [36]. Mutant third instar larvae lacking EGG were recovered from a heterozygous stock carrying the *egg^10.1-1a^* allele [21] over a GFP balancer by selecting for larvae lacking GFP.

### Cell lines

S2-DRSC cells (stock #181) and ML-DmBG3-c2 cells (stock #68) were obtained from the Drosophila Genome Resource Center. Both cell lines are grown at 25°C with 70% humidity according to modENCODE protocols. S2-DRSC cells were grown to a density from 10^6^ to 10^7^ cells/ml in Schneider's media supplemented with 10% FCS (fetal calf serum), and ML-DmBG3-c2 cells were grown to a density from 2×10^6^ to 1.2×10^7^ cells/ml in Schneider's media supplemented with 10% FCS and 10 µg/ml insulin.

### Antibody characterization

All antibodies used for ChIP experiments were characterized using immunoblotting or immunofluorescence to ensure the specificity of the antibody to recognize the histone modification or chromosomal protein in question. Validation protocols for histone antibodies were described in detail in a recent article and consisted of a test for cross-reactivity with non-histone Drosophila proteins as well as a test for modification specificity [63]. Other chromosomal proteins were tested by two methods, immunoblotting or immunofluorescence, to check for cross-reactivity with non-target proteins. By western blot analysis, an antibody meeting the following two criteria was considered passed: 1) a band of the correct size was detected in the wildtype sample, accounting for more than 50% of the total signal in the lane; 2) the intensity of the specific band decreases to less than 50% in mutants or knockdown samples. If immunoblots were unsuccessful, immunofluorescence was used as a characterization measure. For immunofluorescence tests, an antibody meeting the following two criteria were considered passed: 1) the immunofluorescence pattern must conform to expectations (for example, nuclear staining for a chromatin protein); 2) no immunofluorescence signal is detected in mutants. Some antibodies were considered validated if their ChIP profiles were consistent with those of a second, validated antibody to the same protein or to a known complex member. Antibody characterization data are part of the metadata provided with each dataset; they are available at www.modENCODE.org.

### Chromatin immunoprecipitation and microarray processing

Protocols for the preparation of formaldehyde cross-linked chromatin from cultured cells, ChIP conditions, and array hybridization conditions are described in a recent article by Kharchenko and colleagues [14]. For all analyses, heterochromatin/euchromatin border positions previously defined by H3K9me2 enrichment were used [15].

### Data analysis

#### Processing of ChIP–chip data

The M-value (log2 ratio of signal intensities between ChIP and input) was calculated for each array dataset. Data normalization and identification of regions (or peaks) with significant enrichment were performed as described in Kharchenko et al [14]. At least two biological replicates were performed for each ChIP profile included in the analysis. The independent biological replicates were considered consistent if their target lists overlapped more than 75% or if the top 40% of the targets in each replicate had more than 80% in common. For correlation and other analyses, 500 bp bins were used to average the enrichment levels. For the heatmap visualization, metagene profiles were obtained with a scaled gene body of 3 kb. The extended regions of +/−2 kb from the TSS and TES were included.

#### Chromatin states model

The five-state chromatin annotations (based on the K-means algorithm) for the heterochromatin regions in BG3 and S2 cells were obtained from [15]. The number of states in this model was derived by combining states with similar enrichment patterns after starting with a higher number of states.

#### Gene expression analysis

RNA-seq data for BG3 cells, S2 cells, and third instar larvae from Cherbas and colleagues were used for this analysis [64]. Reads Per Kilobase of exon model per Million mapped reads (RPKM) was calculated for each gene. Based on the distributions of expression levels, genes with an log10(RPKM+1)>0.6 were considered expressed for BG3 and S2 cells; the threshold was log10(RPKM+1)>0.4 for third instar larval data. This definition of expressed genes was used throughout the paper, e.g. in metagene analyses.

#### Polymerase pausing analysis

For GRO-seq analysis, the pausing index (PI) was calculated according to the method developed by Larschan and colleagues [26], defining the PI of a gene as the ratio of signal at the 5′end (first 500 bp) to the first 25% of the remaining gene body. For our analysis, genes were divided into three groups: euchromatin, pericentric heterochromatin, and chromosome 4 using the border positions for S2 cells defined by Riddle et al. [15]. A PI threshold value of 10 was used. To confirm these findings, we also estimated occurrence of pausing from ChIP-chip data using the PI proposed by Zeitlinger and colleagues [31], defined as the ratio between the maximum enrichment value around TSS (+/−300 bp) and the medium enrichment values over the gene body (600 bp downstream of TSS to the end of the gene) (Table S3B). Genes with a PI>4 are considered paused by this method. Genes shorter than 500 bp as well as overlapping genes were excluded from the analysis. Significance was determined using a permutation test. To compare method 1 and method 2, the per transcript data from method 1 was converted to per gene data, and overlap was estimated by comparing the top scoring 1,000, 1,500, and 2,000 genes. The overlap is 41% for 1,000 genes, 46% for 1,500 genes, and 50% for 2,000 genes.

For GO analysis, we used the GOToolBox (http://genome.crg.es/GOToolBox/) to compare the 76 chromosome 4 genes with GO annotation to the whole genome reference [65]. A hypergemetrical test with Bonferroni correction was used to determine significance. For motif analysis, the fraction of promoters including a pause button motif (KCGRWCG) [29] was determined using CisGenome [66] with a window of +/−60 bp around the TSS. Significance was determined using a permutation test. For the TRL (GAGA), inverted TRL, and Inr motifs, a window of +/−200 bp around the TSS was used. The T_m_ analysis was carried out as described by Nechaev and colleagues [33].

#### Mutant analysis

Differential gene expression comparing HP1a depletion to wildtype was performed using RNA-seq data (GEO accession GSE39083). RNA was prepared from third instar larvae using Trizol according the manufacturer's recommendations. rRNA-depleted cDNA libraries suitable for Illumina sequencing were prepared and sequencing was carried out by the Genome Technology Access Center (GTAC) at Washington University. FPKM (Fragment per Per Kilobase of exon model per Million mapped reads) values for each gene were calculated using the output from CuffLinks [67]. The cutoff value between expressed/silent genes was log2(FPKM+1) = 1.4. To obtain statistical significances of gene expression changes, we used several independent tools: BaySeq [68], EdgeR [69], and DESeq [70]. The set of genes detected in all analyses as significant was used for analysis. Pausing index for HP1a and POF mutants was calculated as described in [31].

To compare enrichment levels of H3K9me2, H3K9me3, POF and HP1a in various mutants with those in wildtype, we normalized the profiles using noise level signals as proposed in [71]. For each profile, the scaling factor was calculated as the ratio of the median absolute deviation to the lagged differences between mutant and WT. This is defined as median|*d^WT^_i_* - median(*d^WT^_i_*)|/median|*d^Mutant^_i_* - median(*d^Mutant^_i_*)|, where *d^WT^_i_* = *x^WT^_i+1_* - *x^WT^_i_*_, ,_ and *x^WT^_i_*, is the log-ratio of the *i*th probe in WT data. Similarly, *d^Mutant^_i_* = *x^Mutant^_i+1_* - *x^Mutant^_i_*, *x^Mutant^_i_*, is the log-ratio of the *i*th probe in mutant data. The M-value profiles were then normalized by the factors. For RNA pol II profiles, we performed quantile normalization.

## Supporting Information

Figure S1 Enrichment of histone marks and chromosomal proteins in S2 cells. A. Enrichment levels for the novel chromatin marks reported here are mapped onto the five main combinatorial chromatin states for heterochromatin as defined in Riddle et al 2011 [15]. Histone marks are shown in panel 1, chromosomal proteins in panel 2. Repeat enrichment and expression status for each state are shown in panel 3. Panel 4 illustrates the relationship of state and gene structure, while panel 5 shows enrichment/depletion for each chromosome arm. B. Karyotype view of the assembled heterochromatic domains defined by the five combinatorial chromatin states in A. State A: grey; state B: green; state C: purple; state D: blue; state E: orange. The enlarged view of chromosome 4 shows the large fraction of sequences associated with transcriptionally active TSS and elongation (states B, C, D).
(PDF)

Figure S2 Domains on chromosome 4 supporting strong reporter expression are under control of the Polycomb/trx system. A. Red panels: Enrichment profiles for HP1a (bottom), PC (Polycomb; middle), and their overlay (top) for S2 cells. Blue panels: Enrichment profiles for HP1a, PC, and their overlay for BG3 cells. Genes are shown below in black. Red triangles mark the four domains that support full *hsp70-white* expression [region 1 near *ci* (2M-1020; 79,754), region 2 at position 436,655 (7M-201), region 3 near *zfh2* (e.g. 2M-371; 522,600), and region 4 within *sv* (4M-1030; 1,119,408) [10], [24]]. X-axis: Position along chromosome 4 in bp (centromere to the left). Y-axis: Smoothed M-values. B. *hsp70-white* reporter lines with variegating eye phenotype are excluded from regions associated with PC, and DNase I hypersensitive sites (DHS) are associated with genes in the PC domains. For *hsp70-w* reporters, red bars denote insertions with red eye phenotype (full expression), while black bars denotes insertions with variegating eyes.
(PDF)

Figure S3 Distribution of chromosomal proteins and histone marks unique to chromosome 4 in S2 cells. Metagene analysis for the enrichment (averaged smoothed M-values, Y-axis) for selected marks is plotted against position relative to the TSS for a 3 kb scaled metagene (bp, X-axis). The enrichment is examined separately for active (left) and repressed (right) genes in three genomic domains, chromosome 4 (top panel), pericentric heterochromatin (middle panel), and euchromatin (bottom panel), with the number of genes for each category illustrated at the right corner.
(PDF)

Figure S4 Chromosome 4 genes exhibit unique chromatin marks compared to genes in heterochromatin and euchromatin in BG3 cells. Same analysis as shown in Figure 3, now with the same number of genes (N) as present on chromosome 4 randomly chosen from heterochromatin (Hetero) and euchromatin (Eu) as controls.
(PDF)

Figure S5 Chromosome 4 genes exhibit unique chromatin marks compared to genes in heterochromatin and euchromatin in S2 cells. Same analysis as shown in Figure S3, now with the same number of genes (N) as present on chromosome 4 randomly chosen from heterochromatin (Hetero) and euchromatin (Eu) as controls.
(PDF)

Figure S6 Heatmap showing the enrichment of select chromosomal proteins and histone marks at genes on chromosome 4, compared to heterochromatin and euchromatin. A. BG3 cells. B. S2 cells. The region around the TSS and TTS (+/−500 bp) is not scaled, while the gene body is scaled. Therefore, only genes longer than 1 kb are considered here. Enrichment is shown in red, depletion in blue. Eu - euchromatin. Hetero - heterochromatin.
(PDF)

Figure S7 Histogram of the expected incidence of RNA pol II pausing on chromosome 4. Permutation analysis shows that the low pausing incidence on chromosome 4 is significantly different from that expected based on the pausing occurrence in euchromatin (A. p<3e-5) and pericentric heterochromatin (B. p<0.00024).
(PDF)

Figure S8 Overlap between genes identified genome-wide as pausing by the GRO-seq analysis and the RNA pol II ChIP-chip analysis. The PI was calculated according to [26] (GRO-seq data; blue) or [31] (RNA pol II ChIP-chip; green).
(PDF)

Figure S9 Gene features in chromosome 4, pericentric heterochromatin, and euchromatin. A. Genes on chromosome 4 are slightly larger than genes in euchromatin and heterochromatin (with a median of 8,001 bp vs. 1,907 bp vs. 1,844 bp). B. Chromosome 4 genes tend to have more exons than genes in euchromatin and pericentric heterochromatin (with a median of 6 vs. 3 vs. 2). C. Expression levels in different genomic domains are similar. Expression magnitude [log10(RPKM+1), Y-axis] is compared between euchromatin, pericentric heterochromatin, and chromosome 4. The expression levels are slightly higher on chromosome 4 compared to euchromatin. D. Longer genes exhibit higher PI, which indicates RNA polymerase is biased toward TSS. The PI was calculated from GRO-seq data [26]. E. Genes with more exons tend to show higher PI. The PI was calculated as in [31] using ChIP-chip data.
(PDF)

Figure S10 Validation of ChIP-chip HP1a and POF enrichment peaks. A. Fraction of HP1a peaks reduced in HP1a mutants (third instar larvae). 96% of peaks are significantly reduced. X-axis: M-value of HP1a peaks in WT; Y-axis: fraction of peaks reduced in the mutants. B. Fraction of POF peaks reduced in POF mutants (third instar larvae). 79% of peaks are significantly reduced. X-axis: M-value of POF peaks in WT; Y-axis: fraction of peaks reduced in the mutants.
(PDF)

Figure S11 Effect of HP1a depletion on RNA pol II pausing index. A. Ratio of the PI in mutants lacking HP1a compared to wildtype. The PI is defined as the ratio between the maximum enrichment value around TSS (+/−300 bp) and the medium enrichment values over the gene body (600 bp downstream of TSS to the end of the gene) [31]. 53 of 74 genes show an increase in PI, indicated by a ratio larger than 1. B. Histogram of RNA pol II level fold changes in HP1a mutants (log 2; average per gene) for genes on chromosomes 2, 3, and X (bottom panel) and genes on chromosome 4 (top panel), illustrating a decrease of RNA pol II levels for chromosome 4 genes. C. Relationship between RNA pol II level changes (Y-axis, in log 2) and expression level changes (X-axis, in log 10) of chromosome 4 genes in HP1a mutants compared to wildtype. Data points with x<0 and y<0 correspond to genes where both RNA pol II and expression levels decrease upon HP1a depletion (67 of 84 genes on chromosome 4; r = 0.4, Pearson correlation coefficient).
(PDF)

Figure S12 HP1a-regulated euchromatic genes respond differently to HP1a depletion than chromosome 4 genes. A. RNA pol II distribution in wildtype (WT) and HP1a mutants is compared for chromosome 4 genes and HP1a-regulated genes identified by Piacentini and colleagues [35]. The promoter region (left) and gene body (right) are examined separately. B. Bar graphs illustrating the same data as in A. C. Expression changes observed in HP1a mutants for HP1a-regulated genes identified by Piacentini et al. compared to chromosome 4 genes. D. Expression changes observed in HP1a mutants comparing chromosome 3R genes to chromosome 4 genes.
(PDF)

Figure S13 Effect of POF depletion on RNA pol II. A. Ratio of the pausing index in *pof* mutants and wildtype. The PI is defined as the ratio between the maximum enrichment value around TSS (+/−300 bp) and the medium enrichment values over the gene body (600 bp downstream of TSS to the end of the gene) [31]. 63 of 74 genes on chromosome 4 show an increased PI. B. Histogram of RNA pol II level fold changes in *pof* mutants (log 2; average per gene) for genes on chromosomes 2, 3, and X (upper) and genes on chromosome 4 (lower), illustrating a decrease of RNA pol II levels for chromosome 4 genes.
(PDF)

Figure S14 Relationship between protein enrichment on chromosome 4 genes and their PI change observed in mutant lacking HP1a. A. Heatmap illustrating protein enrichment (red – enrichment, blue – depletion) for genes are ordered by their PI changes in HP1a mutants (in grey below enrichment panel). Data source: Third instar larvae. B. Histogram illustrating the correlation between the enrichment of select proteins at chromosome 4 genes with the PI change in HP1a mutants.
(PDF)

Figure S15 Residual HP1a in *pof* and *egg* mutants is associated with repeated sequences. A. Histogram showing the distance between residual HP1a and repeats in *pof* mutants. The observed distance is significantly smaller than the expected distance based on permutation analysis (38 bp vs. 132 bp, p<0.001). B. Histogram illustrating the distance between residual HP1a and repeats in *egg* mutants. The observed distance is significantly smaller than the expected distance based on permutation analysis (19 bp vs. 135 bp, p<0.001).
(PDF)

Figure S16 Heterochromatic marks are maintained at wildtype levels in the ∼70 kb of assembled sequence adjacent to the centromere in larvae lacking POF (*pof^D119^*). ChIP results from wildtype and mutant larvae are compared. The first panel shows RNA-seq data from wildtype larvae. X-axis: position along chromosome 4 in bp; Y-axis: ChIP enrichment for HP1a (top), H3K9me2 (middle), and H3K9me3 (bottom).
(PDF)

Table S1 Description of ChIP datasets. Overlap of top 40% peaks – for the replicate experiments, overlap between the top 40% of peaks is calculated, with peaks calls based on p-value enrichment scores. Overlap based on size-adjusted threshold – for replicate experiments, overlap between the peaks is determined with peaks calls based on p-value enrichment scores and adjusted for the number of peaks called in each experiment. Correlation coefficient r – correlation coefficient is calculated from the log10 p-value enrichment scores of the two replicate experiments. When more than two replicate datasets were available, the average correlation coefficient is reported.
(XLS)

Table S2 Description of ChIP datasets from mutants. Overlap of top 40% peaks – for the replicate experiments, overlap between the top 40% of peaks is calculated, with peaks calls based on p-value enrichment scores. Overlap based on size-adjusted threshold – for replicate experiments, overlap between the peaks is determined with peaks calls based on p-value enrichment scores and adjusted for the number of peaks called in each experiment. Correlation coefficient r – correlation coefficient is calculated from the log10 p-value enrichment scores of the two replicate experiments. When more than two replicate datasets were available, the average correlation coefficient is reported.
(XLSX)

Table S3 Polymerase pausing is rare on chromosome 4. Genes with paused RNA pol II have been identified by two methods. A. Polymerase pausing determined by GRO-seq analysis in S2 cells (Method 1). B. Genes with paused polymerase are identified solely based on ChIP-chip mapping of RNA pol II ([31]; Method 2). The percentage of paused genes is reported relative to the number of genes associated with RNA pol II. Note: In the table below, in A, the analysis is carried out on a per transcript basis. Thus, the total number of “genes” is inflated as some genes have multiple different transcripts and TSSs.
(DOCX)

Table S4 Developmental control genes are not depleted among chromosome 4 genes. GO term analysis demonstrates that the following terms are significantly enriched among chromosome 4 genes (p<0.05). No terms are significantly depleted.
(DOCX)

Table S5 The frequency of GAGA factor motifs, but not the pause button sequence, is underrepresented in chromosome 4 promoters. PB - Pause button. GAGA factor (TRL) binding motif – TRL motif. Inverted GAGA factor binding motif – iTRL motif.
(DOCX)

Table S6 The average T_m_ of 9-mer sequences downstream of TSSs on chromosome 4 is significantly lower than on other chromosomes. The minimum (min.), median, mean, and maximum (max.) melting temperature (T_m_, °C) for 9-mer sequences in the first 100 bp downstream of all unique TSSs in the *D. melanogaster* genome are compared by chromosome arm.
(DOCX)

Table S7 HP1a and H3K9me2/3 enriched regions on chromosome 4 in wildtype, *pof^D119^*, *Su(var)205*, and *egg^10.1a^* mutant third instar larvae. Significantly enriched regions based on smoothed M-value profiles (FDR = 1e-3) are compared between wildtype and mutant. Note that the magnitude of the enriched peaks is not considered. POF+ region: POF enriched region in wildtype; POF- region: No POF enrichment in wildtype. HP1a+ region: HP1a enriched region in wildtype; HP1a- region: No HP1a enrichment in wildtype.
(DOCX)
