# Supplementary figures and images for: Enrichment of HP1a on Drosophila Chromosome 4 Genes Creates an Alternate Chromatin Structure Critical for Regulation in this Heterochromatic Domain
Source: PLoS Genet. 2012 Sep 20;8(9):e1002954. doi: 10.1371/journal.pgen.1002954 (PMC3447959; doi:10.1371/journal.pgen.1002954)

# Figure S1

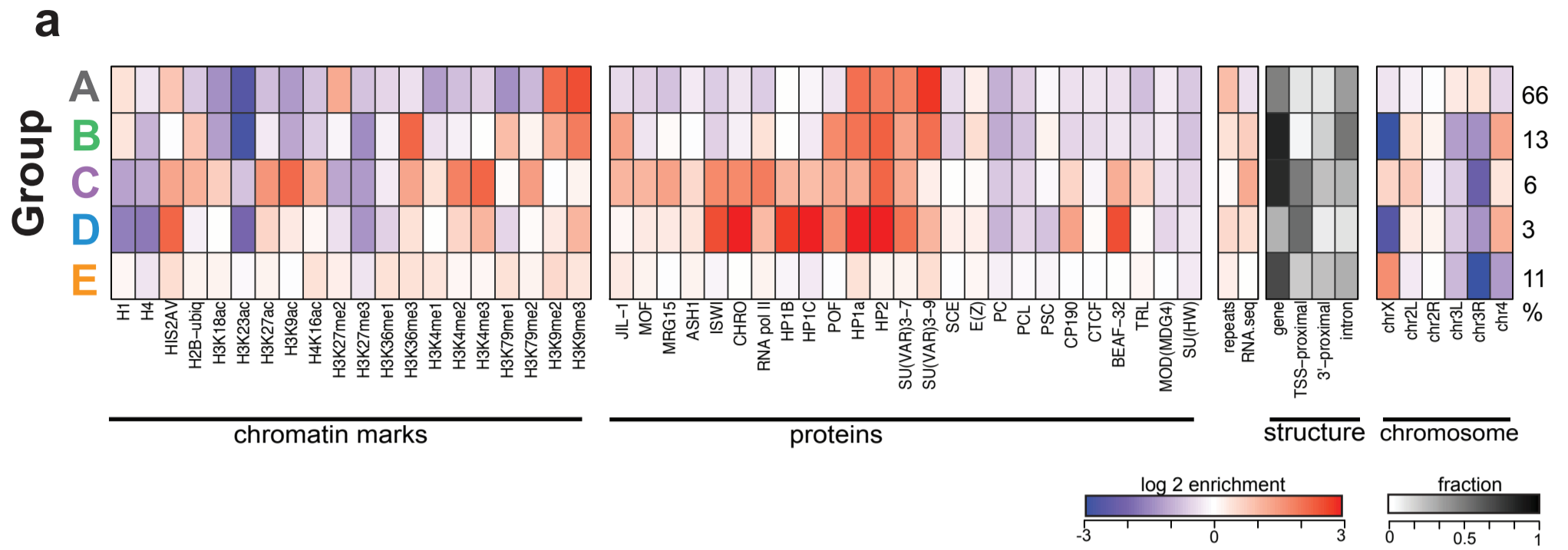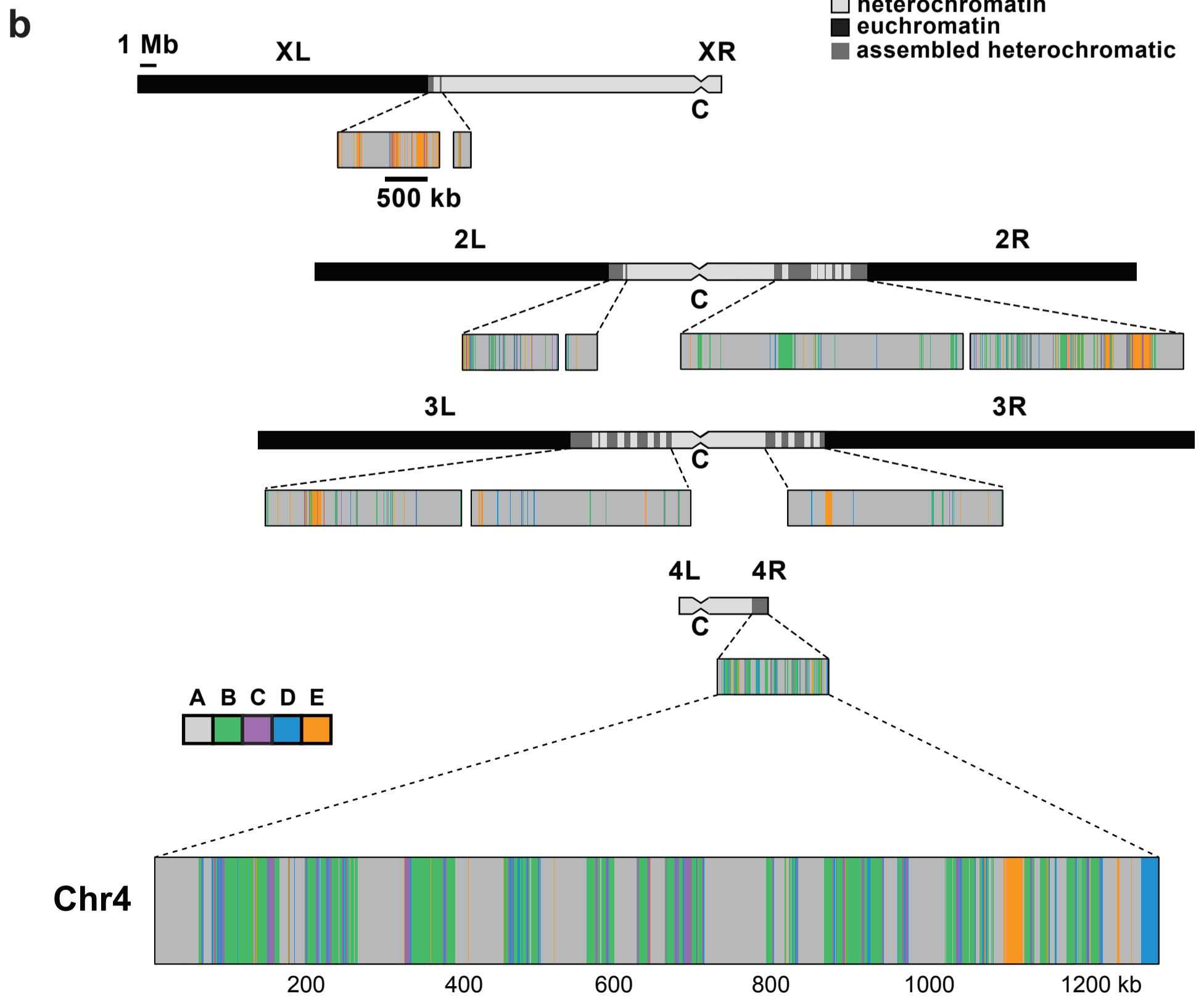

Supplement: Figure S1 — Enrichment of histone marks and chromosomal proteins in S2 cells. A. Enrichment levels for the novel chromatin marks reported here are mapped onto the five main combinatorial chromatin states for heterochromatin as defined in Riddle et al 2011 [15]. Histone marks are shown in panel 1, chromosomal proteins in panel 2. Repeat enrichment and expression status for each state are shown in panel 3. Panel 4 illustrates the relationship of state and gene structure, while panel 5 shows enrichment/depletion for each chromosome arm. B. Karyotype view of the assembled heterochromatic domains defined by the five combinatorial chromatin states in A. State A: grey; state B: green; state C: purple; state D: blue; state E: orange. The enlarged view of chromosome 4 shows the large fraction of sequences associated with transcriptionally active TSS and elongation (states B, C, D). (PDF) [file pgen.1002954.s001.pdf]

**a**

# Figure S2

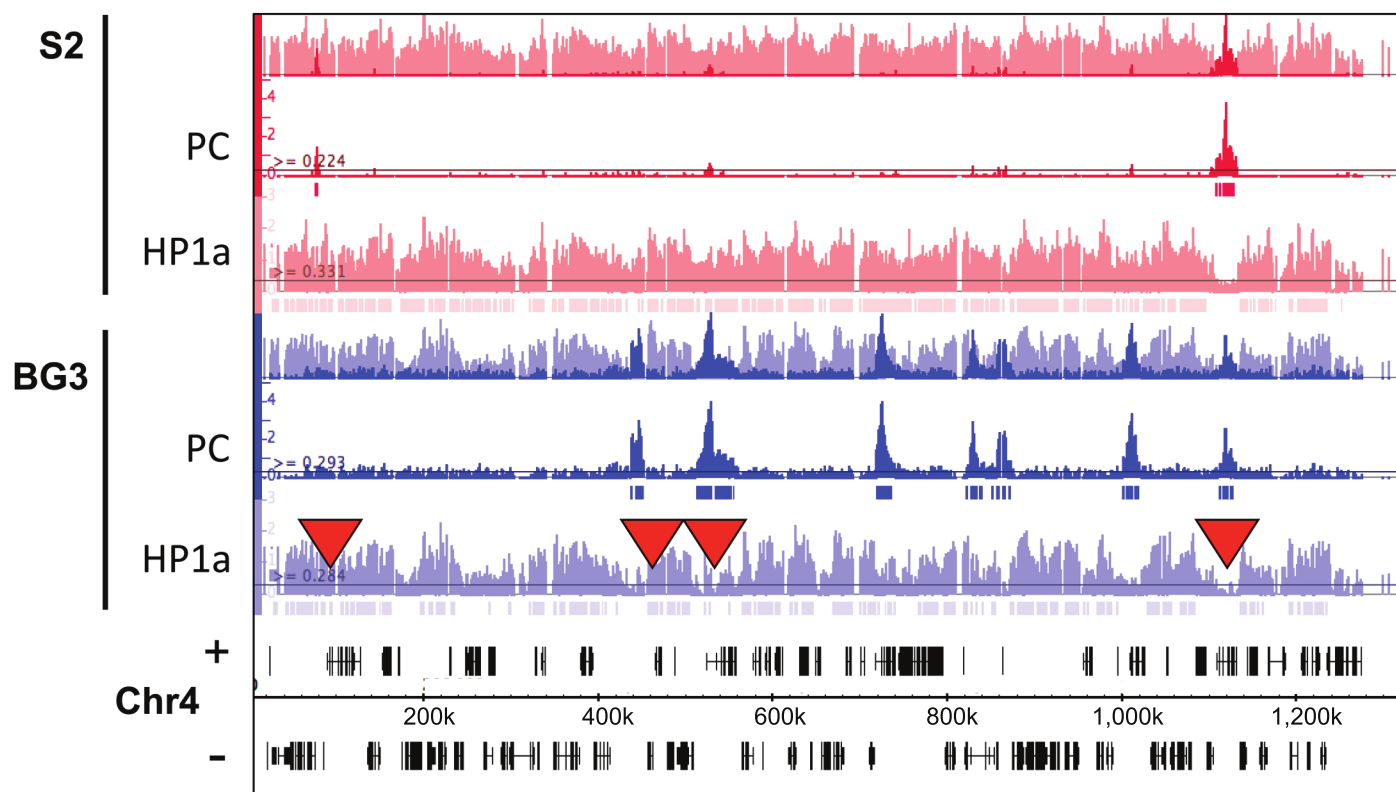**b**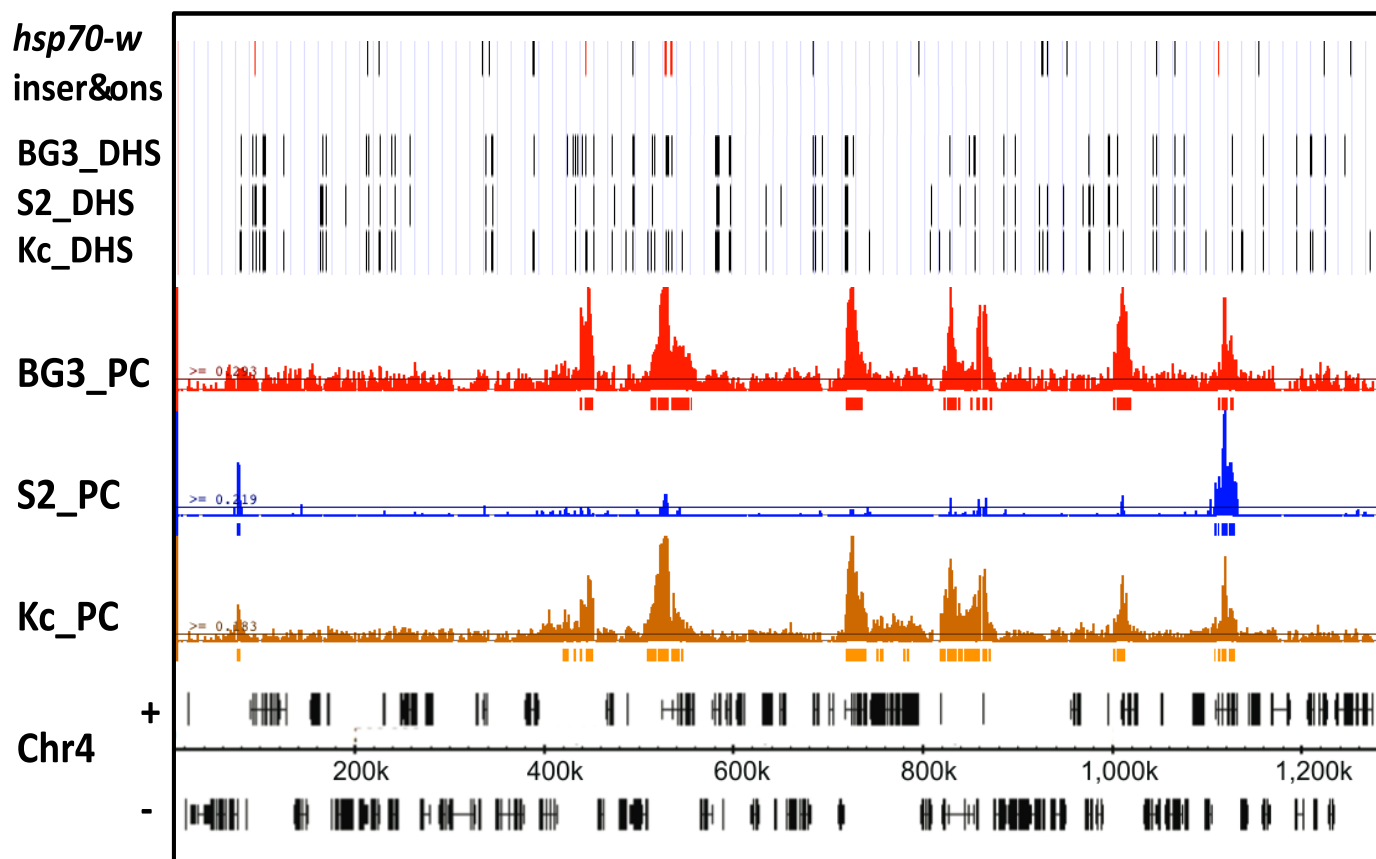

Supplement: Figure S2 — Domains on chromosome 4 supporting strong reporter expression are under control of the Polycomb/trx system. A. Red panels: Enrichment profiles for HP1a (bottom), PC (Polycomb; middle), and their overlay (top) for S2 cells. Blue panels: Enrichment profiles for HP1a, PC, and their overlay for BG3 cells. Genes are shown below in black. Red triangles mark the four domains that support full hsp70-white expression [region 1 near ci (2M-1020; 79,754), region 2 at position 436,655 (7M-201), region 3 near zfh2 (e.g. 2M-371; 522,600), and region 4 within sv (4M-1030; 1,119,408) [10], [24]]. X-axis: Position along chromosome 4 in bp (centromere to the left). Y-axis: Smoothed M-values. B. hsp70-white reporter lines with variegating eye phenotype are excluded from regions associated with PC, and DNase I hypersensitive sites (DHS) are associated with genes in the PC domains. For hsp70-w reporters, red bars denote insertions with red eye phenotype (full expression), while black bars denotes insertions with variegating eyes. (PDF) [file pgen.1002954.s002.pdf]

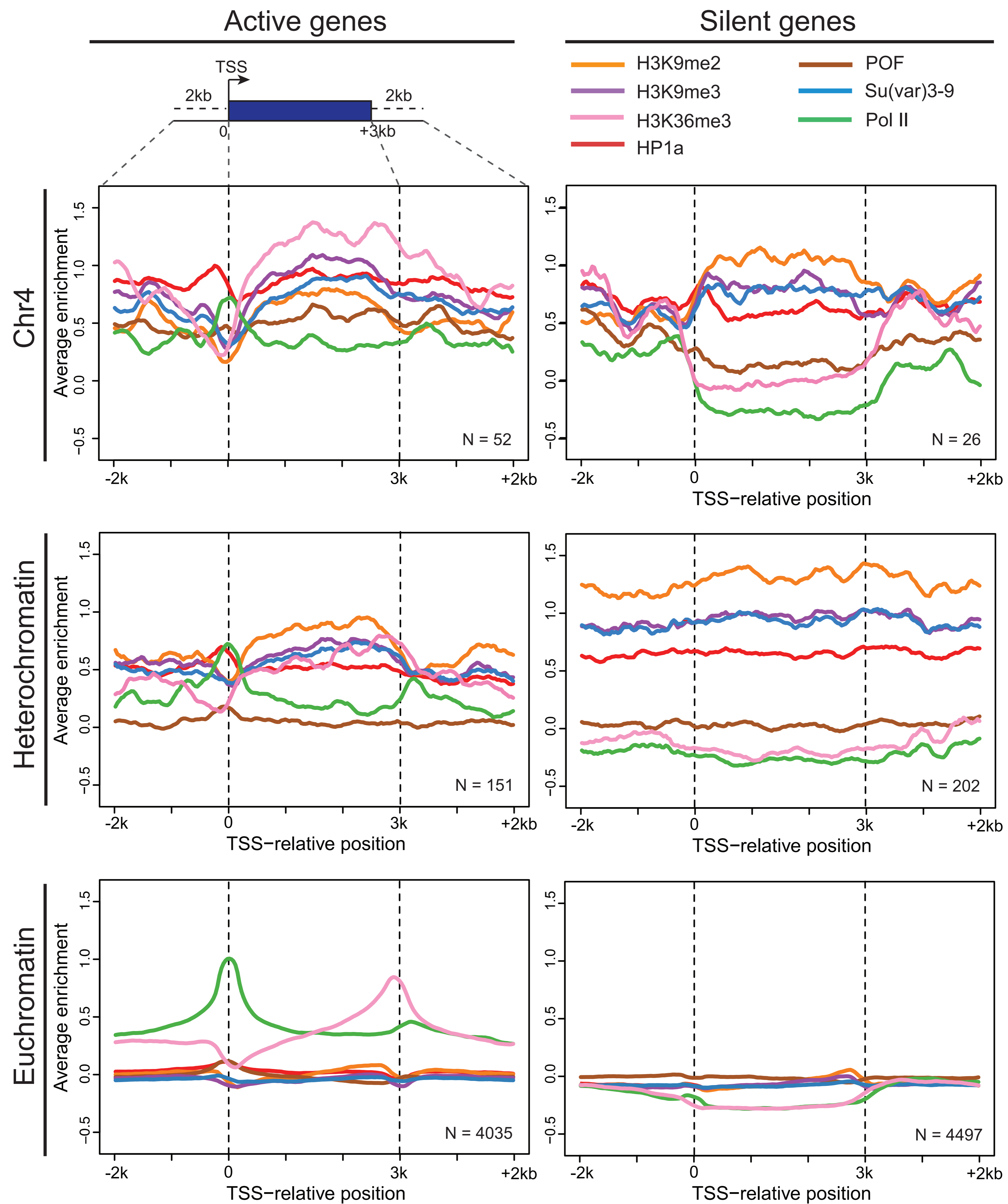

Figure S3

Supplement: Figure S3 — Distribution of chromosomal proteins and histone marks unique to chromosome 4 in S2 cells. Metagene analysis for the enrichment (averaged smoothed M-values, Y-axis) for selected marks is plotted against position relative to the TSS for a 3 kb scaled metagene (bp, X-axis). The enrichment is examined separately for active (left) and repressed (right) genes in three genomic domains, chromosome 4 (top panel), pericentric heterochromatin (middle panel), and euchromatin (bottom panel), with the number of genes for each category illustrated at the right corner. (PDF) [file pgen.1002954.s003.pdf]

**a** BG3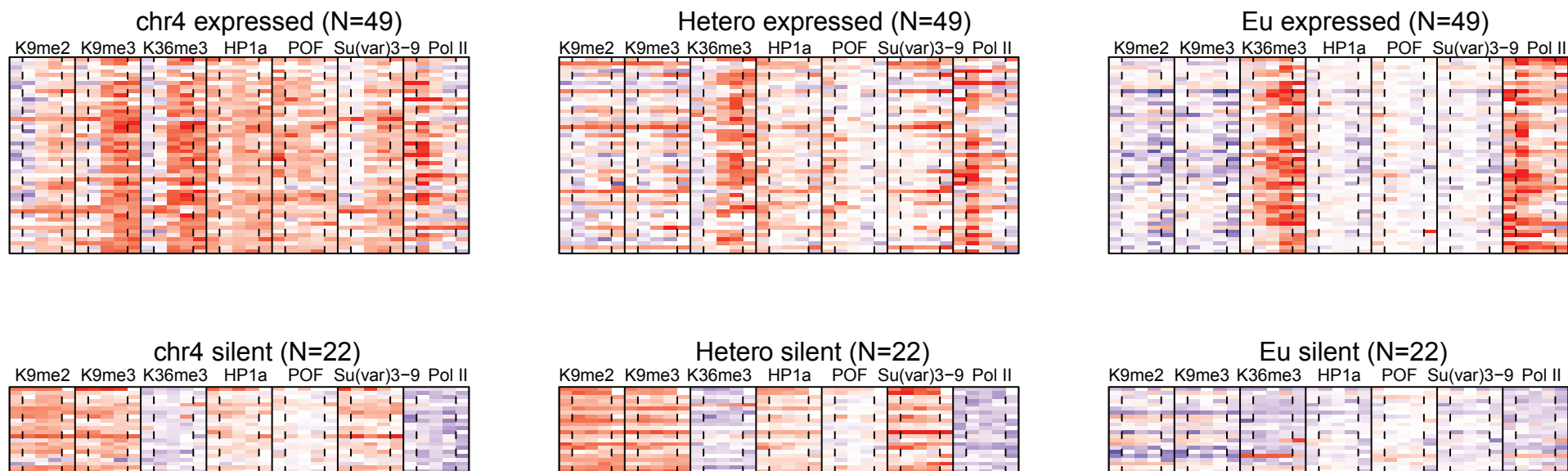**b** S2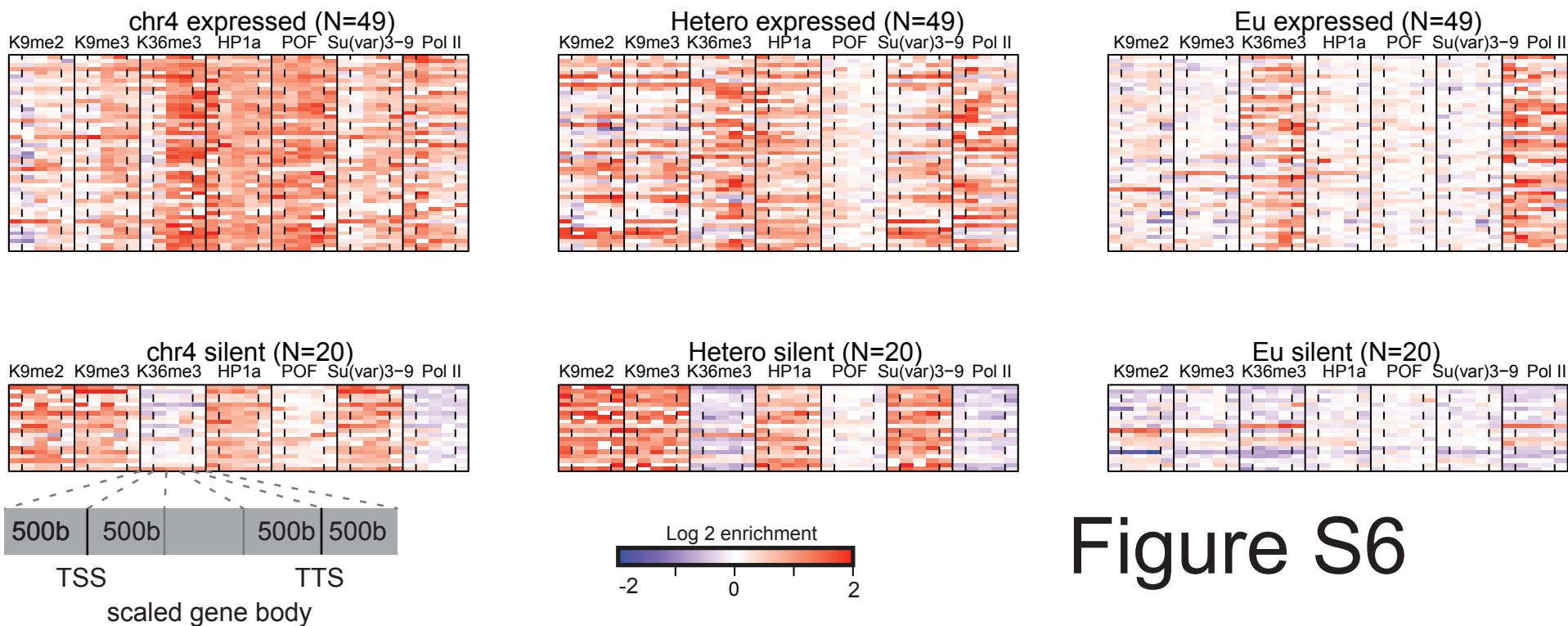**Figure S6**

Supplement: Figure S6 — Heatmap showing the enrichment of select chromosomal proteins and histone marks at genes on chromosome 4, compared to heterochromatin and euchromatin. A. BG3 cells. B. S2 cells. The region around the TSS and TTS (+/−500 bp) is not scaled, while the gene body is scaled. Therefore, only genes longer than 1 kb are considered here. Enrichment is shown in red, depletion in blue. Eu - euchromatin. Hetero - heterochromatin. (PDF) [file pgen.1002954.s006.pdf]

**GRO-seq**

**PolII ChIP**

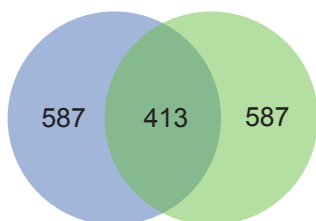

N=1000

41% ( $p < 1e-16$ )

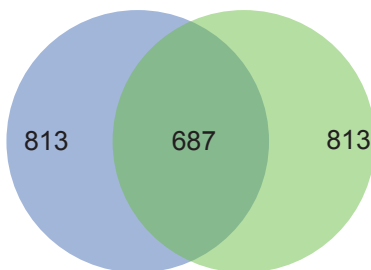

N=1500

46% ( $p < 1e-16$ )

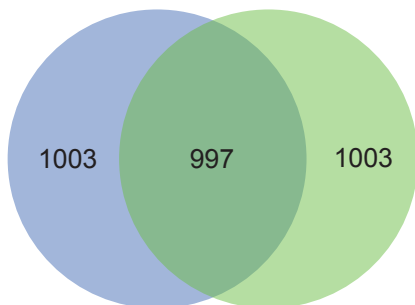

N=2000

50% ( $p < 1e-16$ )

**Figure S8**

Supplement: Figure S8 — Overlap between genes identified genome-wide as pausing by the GRO-seq analysis and the RNA pol II ChIP-chip analysis. The PI was calculated according to [26] (GRO-seq data; blue) or [31] (RNA pol II ChIP-chip; green). (PDF) [file pgen.1002954.s008.pdf]

# Figure S9

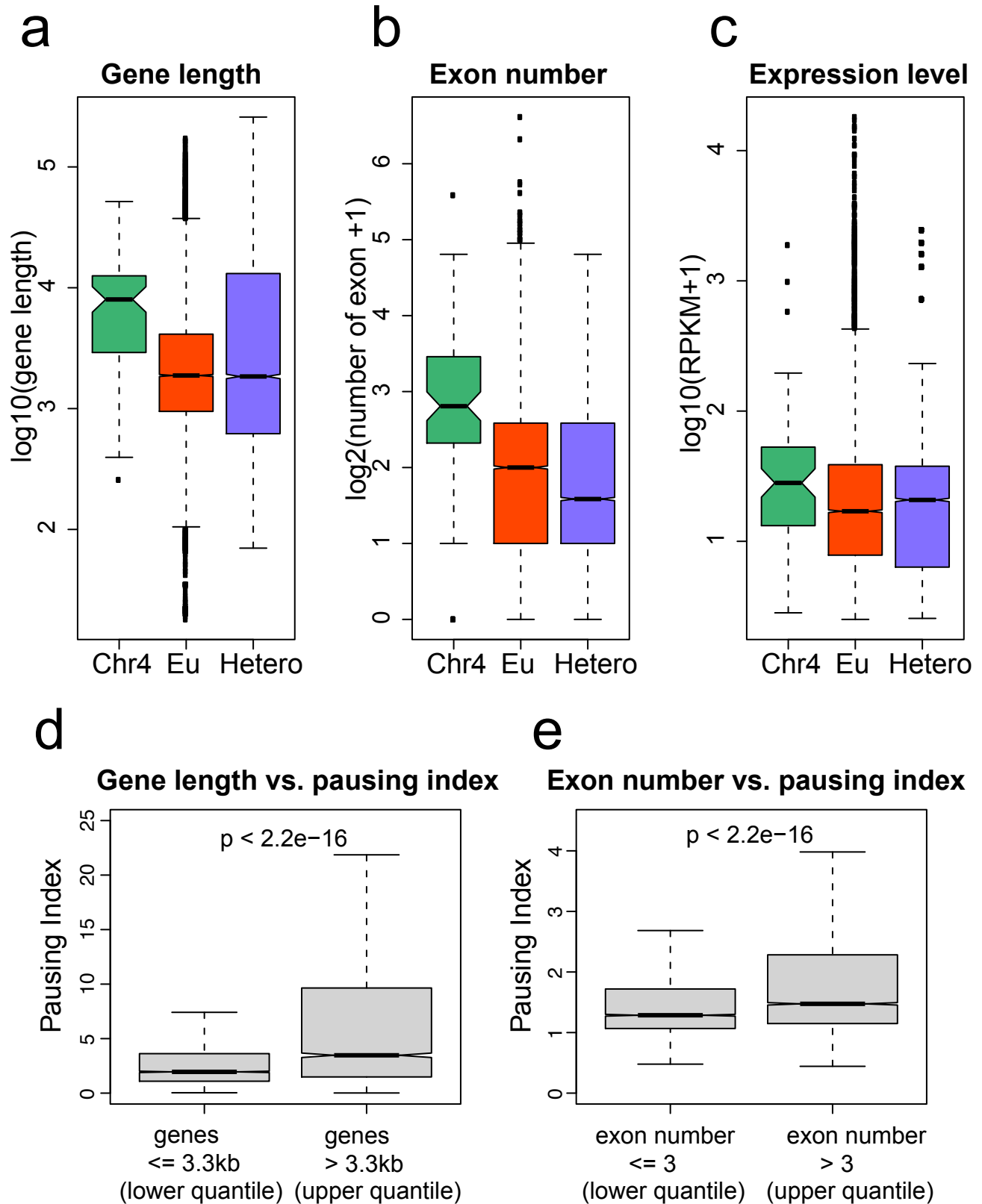

Supplement: Figure S9 — Gene features in chromosome 4, pericentric heterochromatin, and euchromatin. A. Genes on chromosome 4 are slightly larger than genes in euchromatin and heterochromatin (with a median of 8,001 bp vs. 1,907 bp vs. 1,844 bp). B. Chromosome 4 genes tend to have more exons than genes in euchromatin and pericentric heterochromatin (with a median of 6 vs. 3 vs. 2). C. Expression levels in different genomic domains are similar. Expression magnitude [log10(RPKM+1), Y-axis] is compared between euchromatin, pericentric heterochromatin, and chromosome 4. The expression levels are slightly higher on chromosome 4 compared to euchromatin. D. Longer genes exhibit higher PI, which indicates RNA polymerase is biased toward TSS. The PI was calculated from GRO-seq data [26]. E. Genes with more exons tend to show higher PI. The PI was calculated as in [31] using ChIP-chip data. (PDF) [file pgen.1002954.s009.pdf]

# Figure S10

**a**

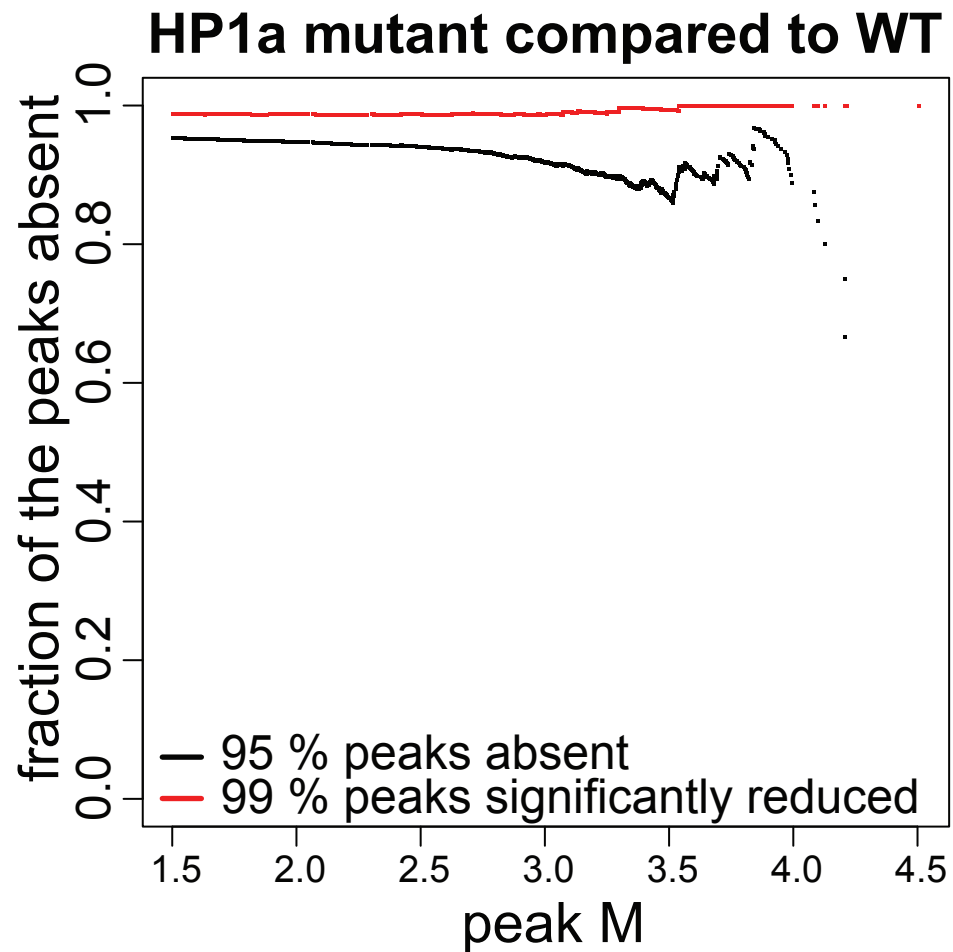

**b**

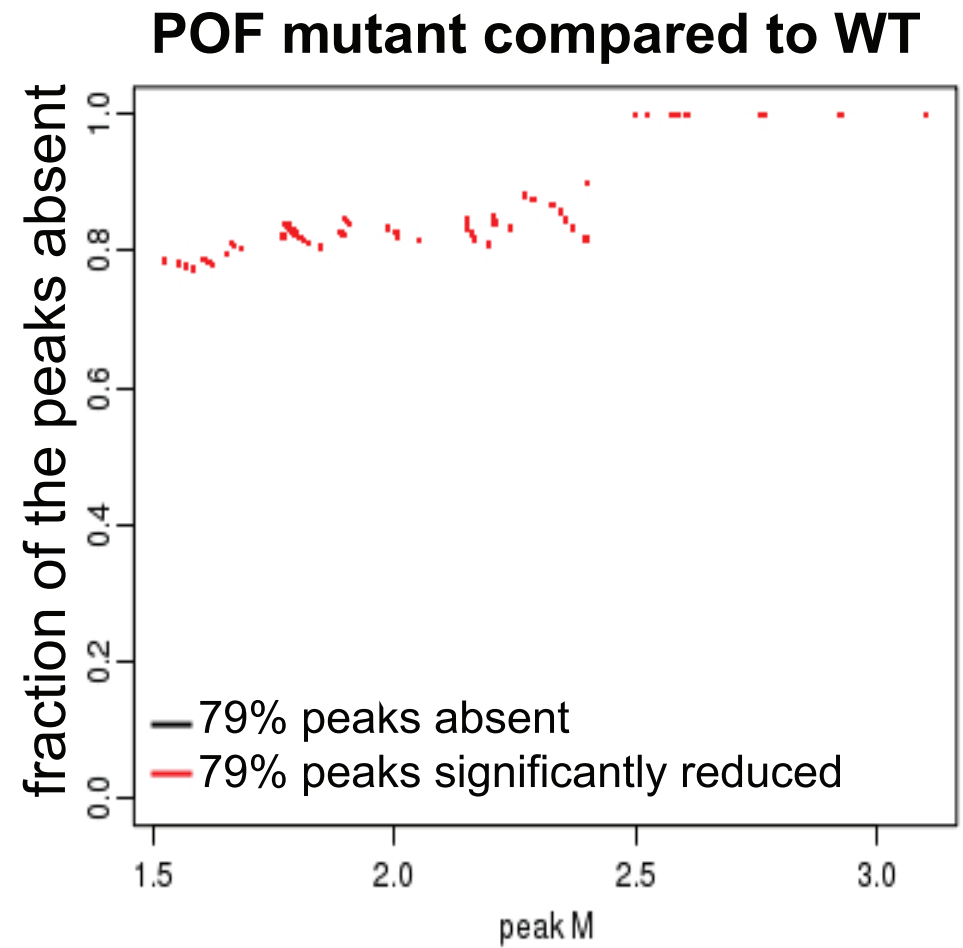

Supplement: Figure S10 — Validation of ChIP-chip HP1a and POF enrichment peaks. A. Fraction of HP1a peaks reduced in HP1a mutants (third instar larvae). 96% of peaks are significantly reduced. X-axis: M-value of HP1a peaks in WT; Y-axis: fraction of peaks reduced in the mutants. B. Fraction of POF peaks reduced in POF mutants (third instar larvae). 79% of peaks are significantly reduced. X-axis: M-value of POF peaks in WT; Y-axis: fraction of peaks reduced in the mutants. (PDF) [file pgen.1002954.s010.pdf]

# Figure S12

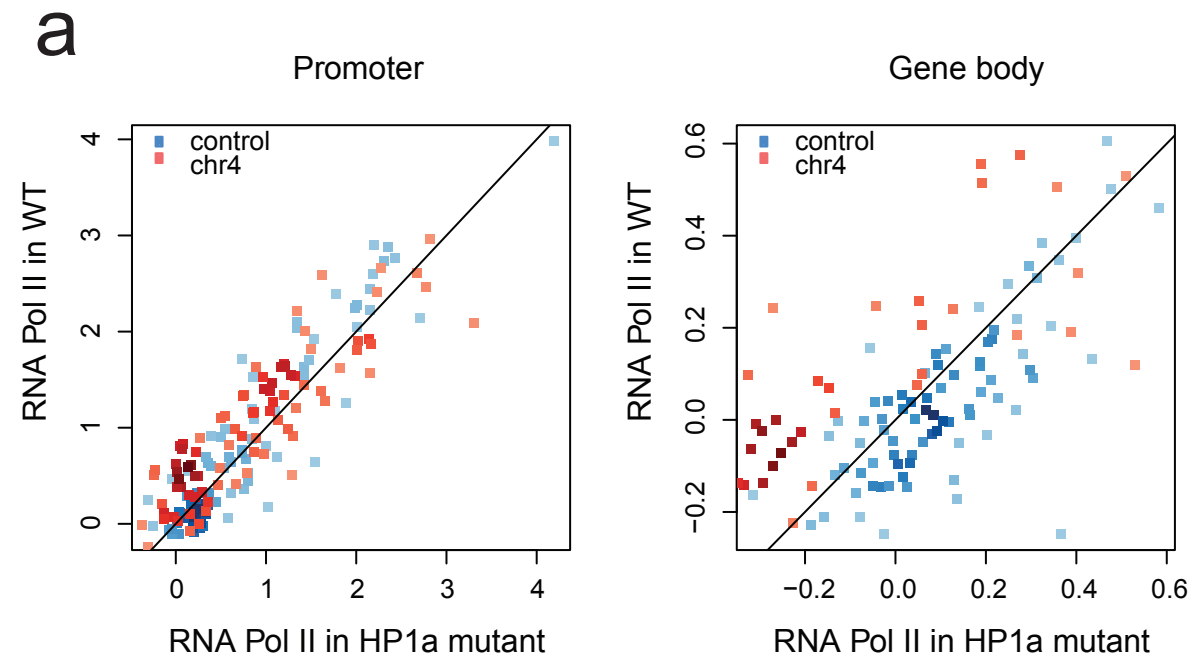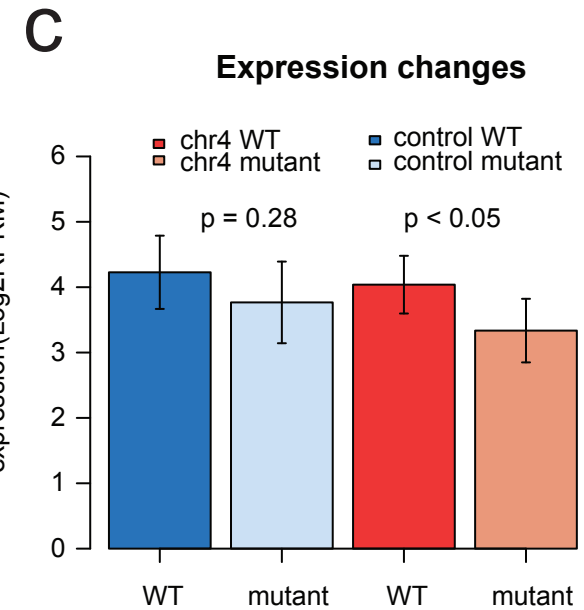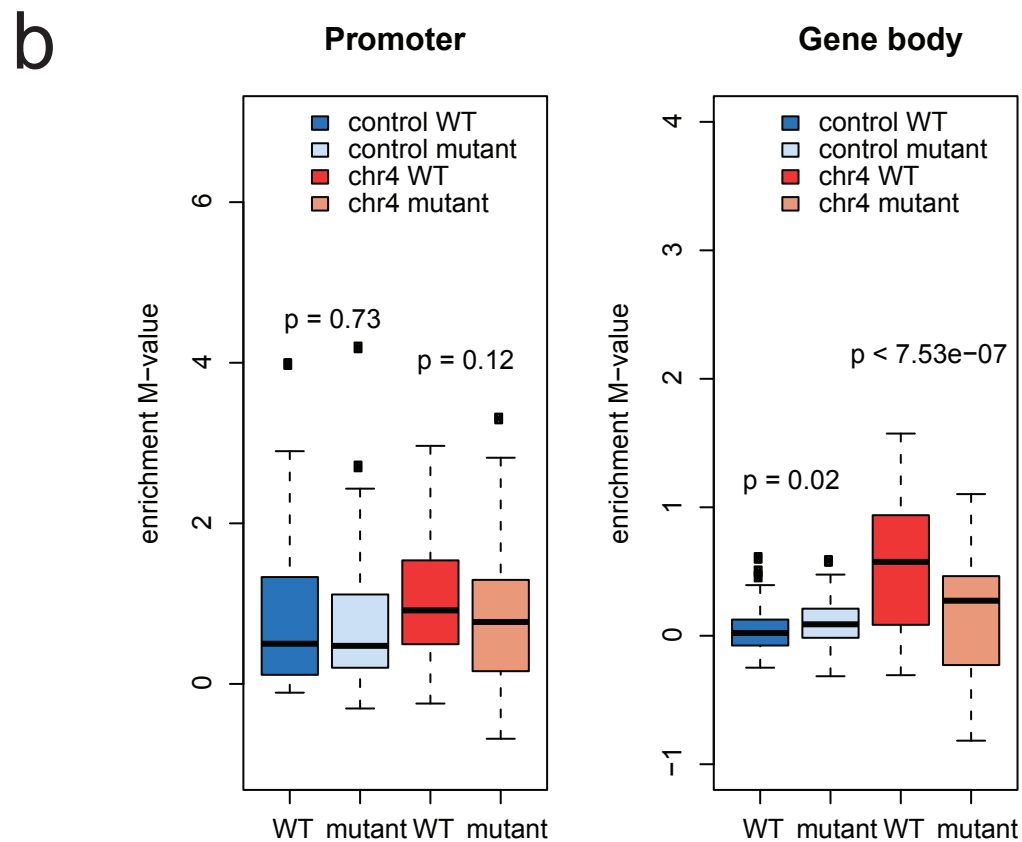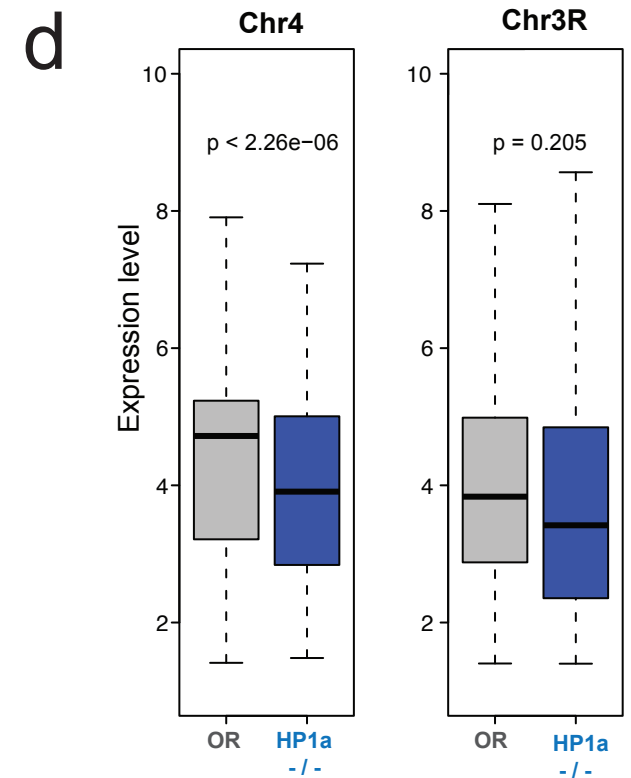

Supplement: Figure S12 — HP1a-regulated euchromatic genes respond differently to HP1a depletion than chromosome 4 genes. A. RNA pol II distribution in wildtype (WT) and HP1a mutants is compared for chromosome 4 genes and HP1a-regulated genes identified by Piacentini and colleagues [35]. The promoter region (left) and gene body (right) are examined separately. B. Bar graphs illustrating the same data as in A. C. Expression changes observed in HP1a mutants for HP1a-regulated genes identified by Piacentini et al. compared to chromosome 4 genes. D. Expression changes observed in HP1a mutants comparing chromosome 3R genes to chromosome 4 genes. (PDF) [file pgen.1002954.s012.pdf]

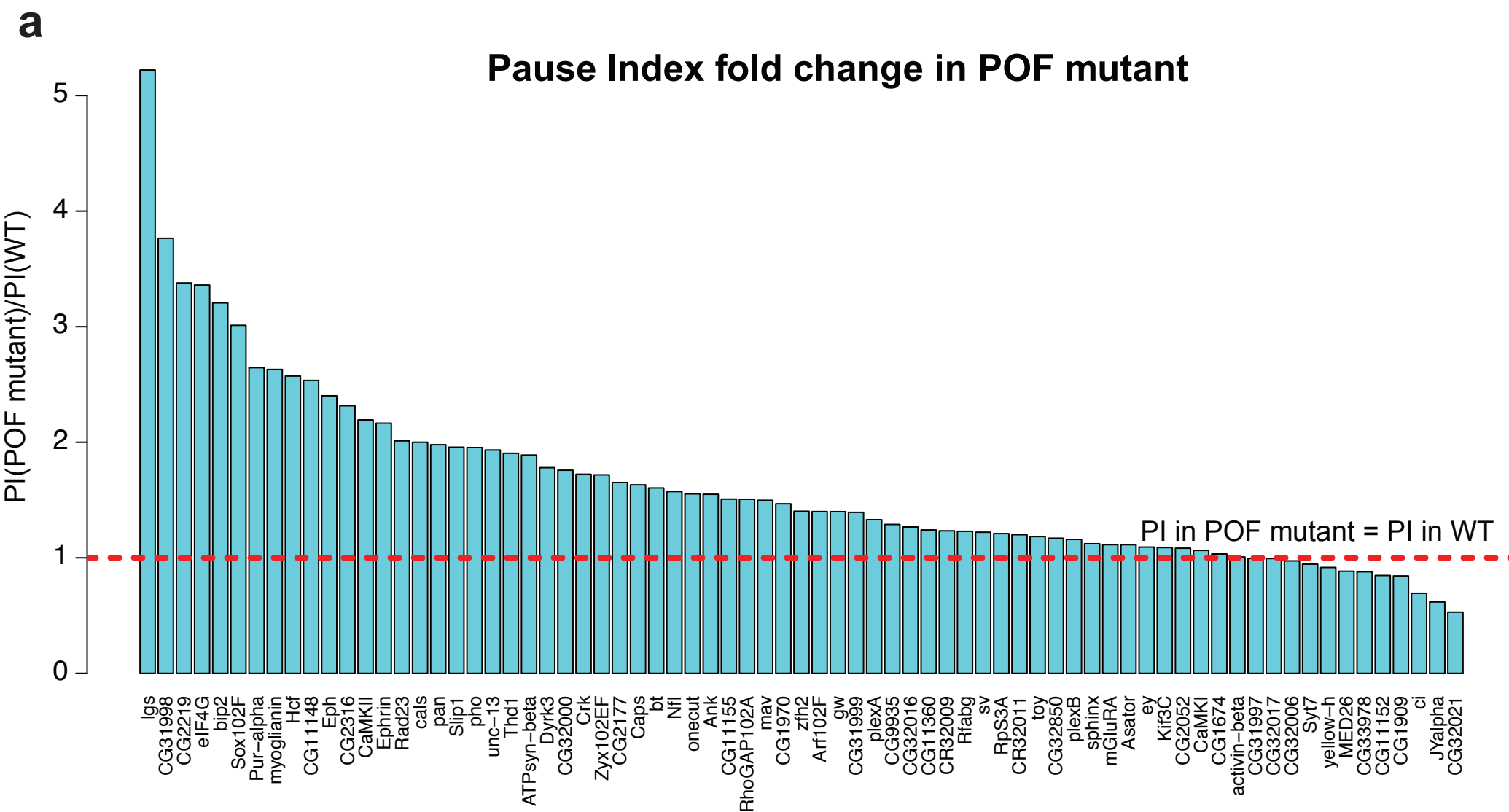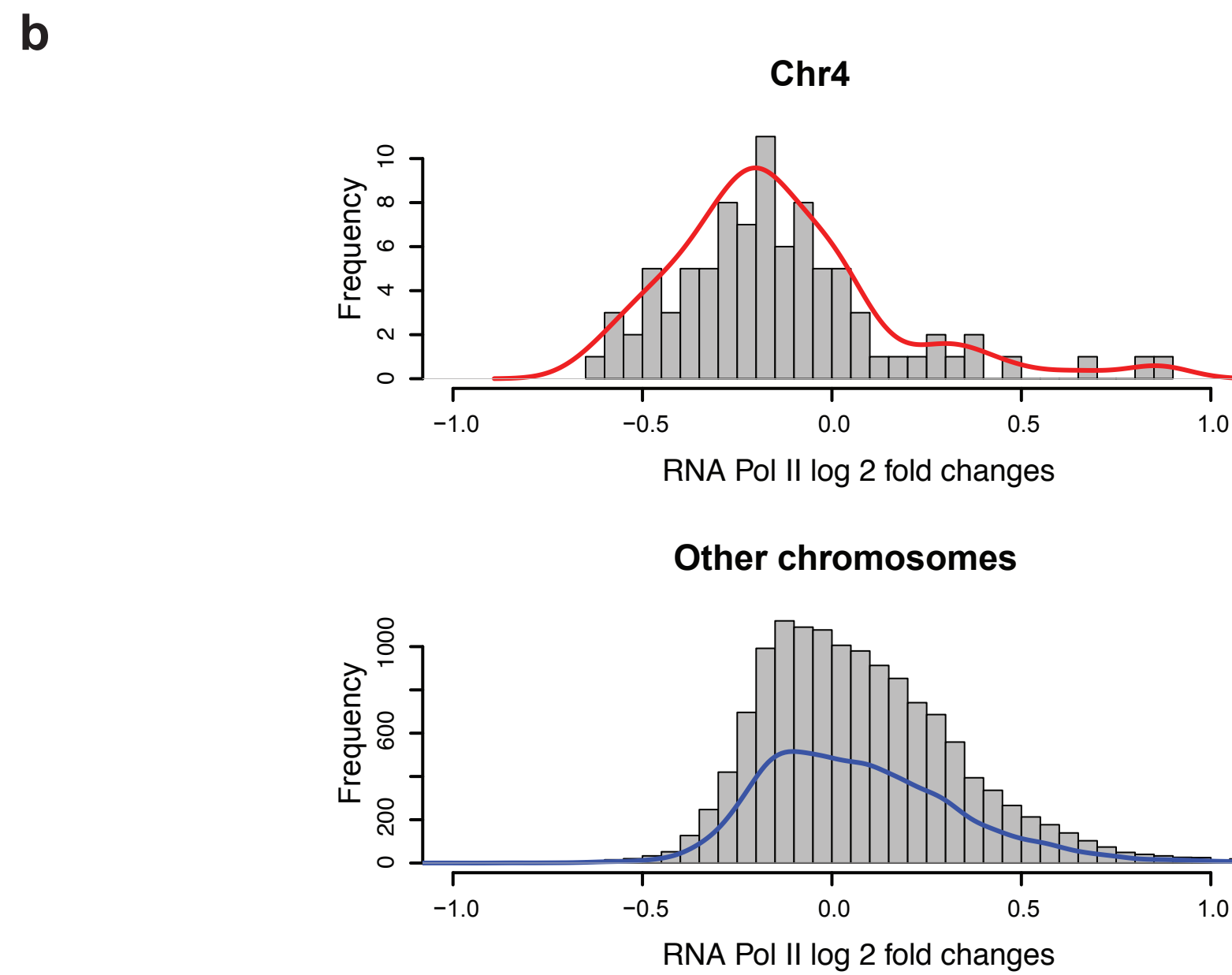

Figure S13

Supplement: Figure S13 — Effect of POF depletion on RNA pol II. A. Ratio of the pausing index in pof mutants and wildtype. The PI is defined as the ratio between the maximum enrichment value around TSS (+/−300 bp) and the medium enrichment values over the gene body (600 bp downstream of TSS to the end of the gene) [31]. 63 of 74 genes on chromosome 4 show an increased PI. B. Histogram of RNA pol II level fold changes in pof mutants (log 2; average per gene) for genes on chromosomes 2, 3, and X (upper) and genes on chromosome 4 (lower), illustrating a decrease of RNA pol II levels for chromosome 4 genes. (PDF) [file pgen.1002954.s013.pdf]

**a**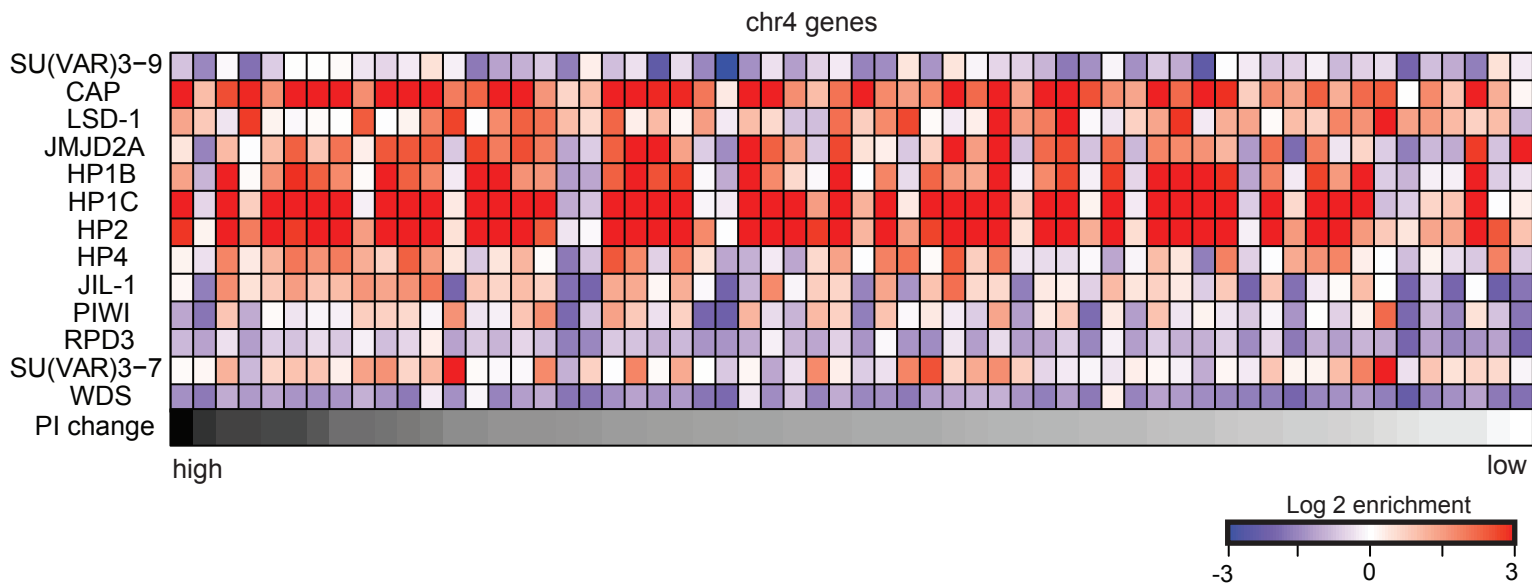**b****correlation with PI changes**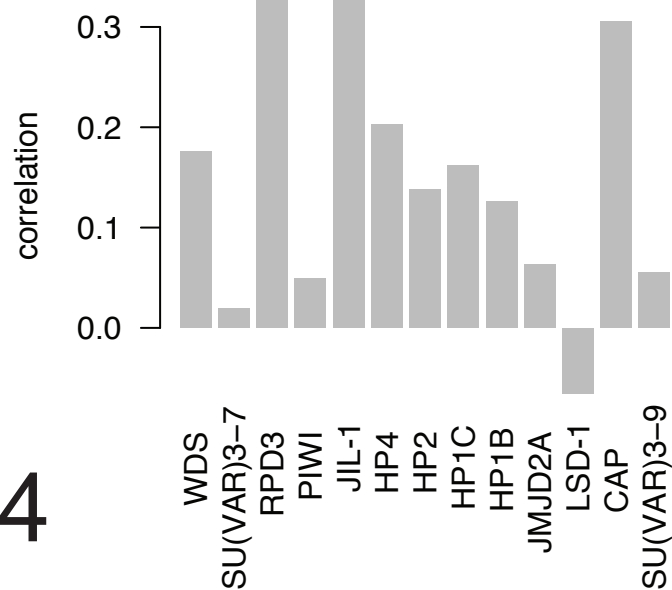**Figure S14**

Supplement: Figure S14 — Relationship between protein enrichment on chromosome 4 genes and their PI change observed in mutant lacking HP1a. A. Heatmap illustrating protein enrichment (red – enrichment, blue – depletion) for genes are ordered by their PI changes in HP1a mutants (in grey below enrichment panel). Data source: Third instar larvae. B. Histogram illustrating the correlation between the enrichment of select proteins at chromosome 4 genes with the PI change in HP1a mutants. (PDF) [file pgen.1002954.s014.pdf]

# Figure S15

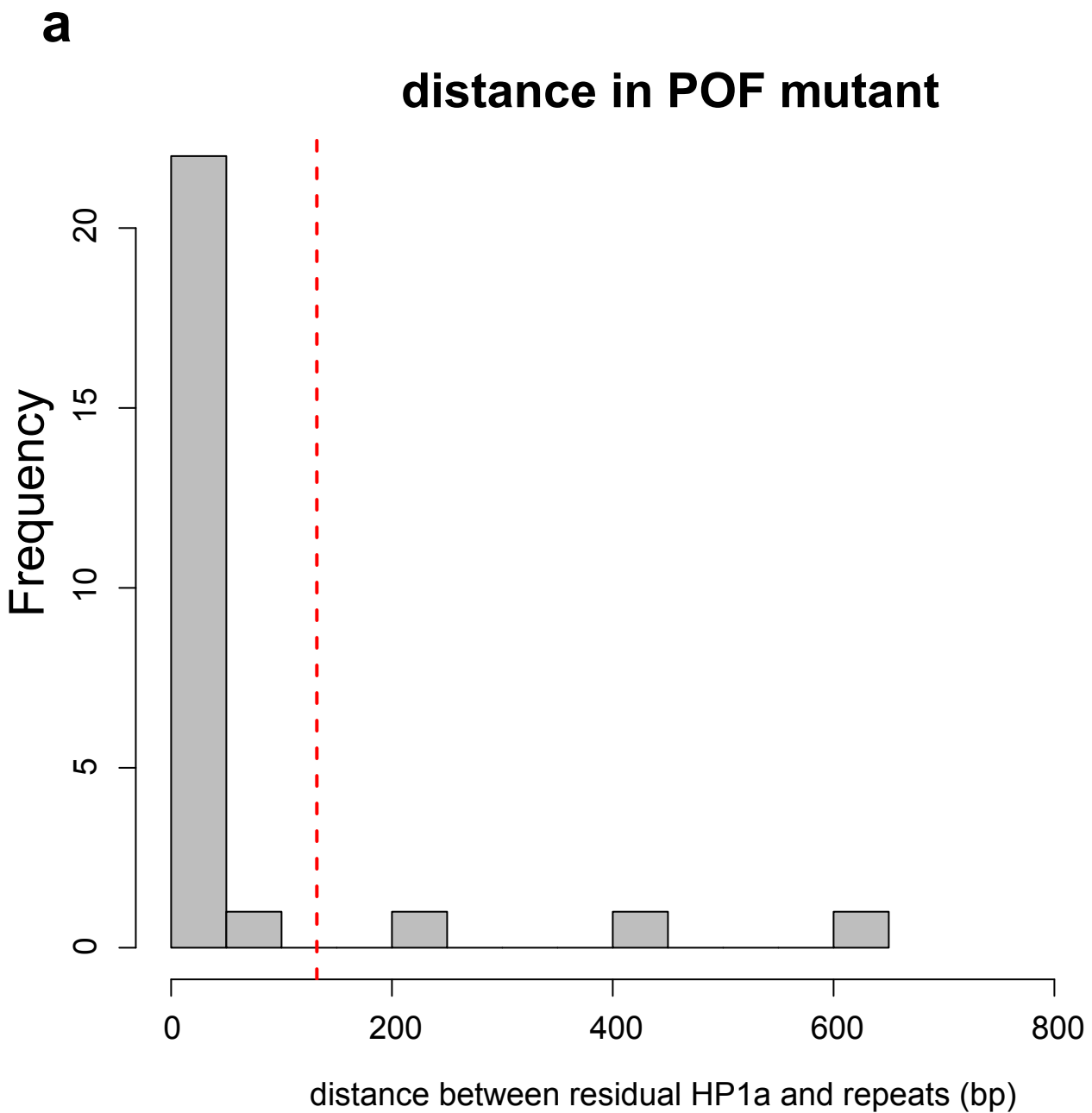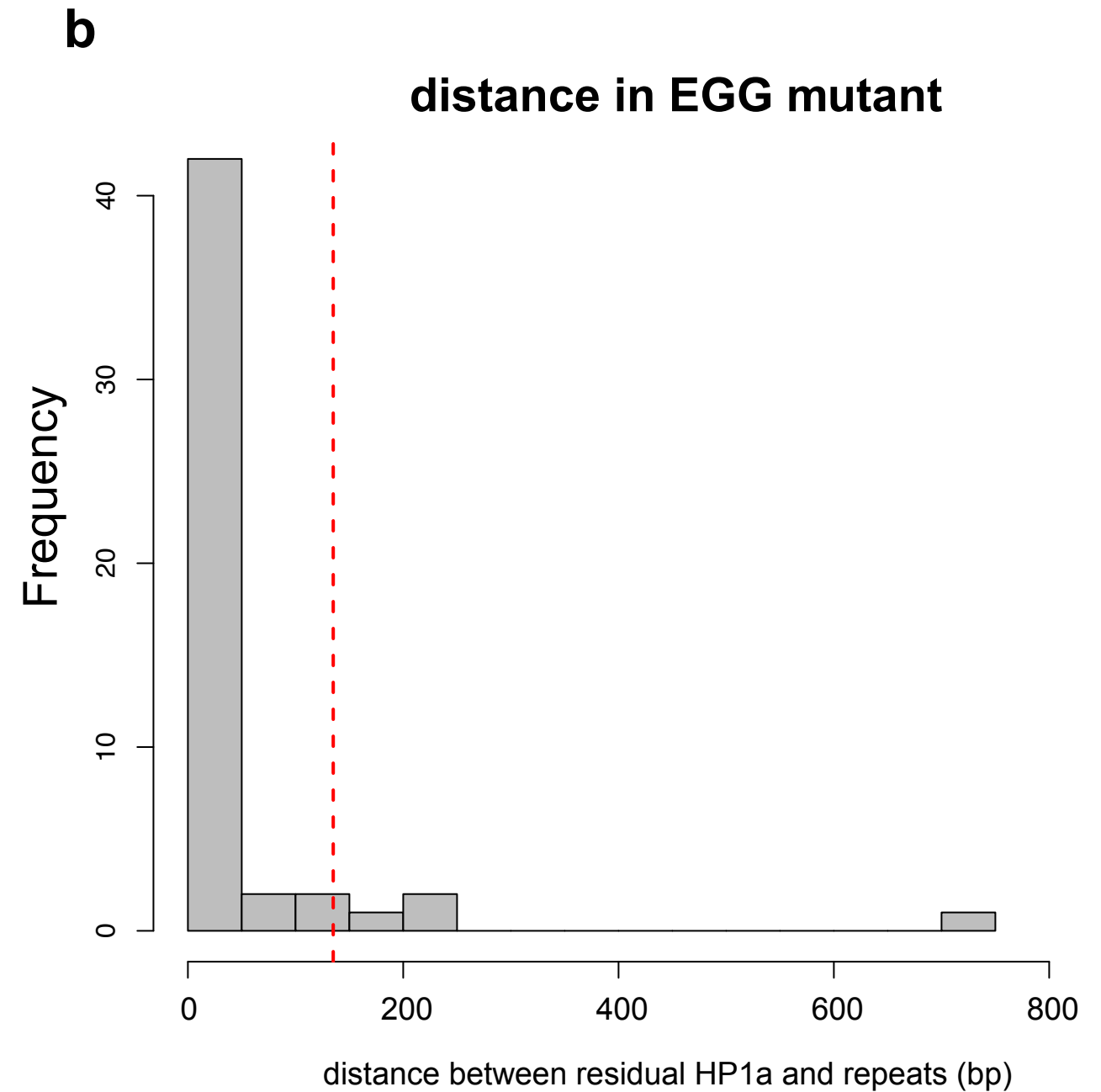

Supplement: Figure S15 — Residual HP1a in pof and egg mutants is associated with repeated sequences. A. Histogram showing the distance between residual HP1a and repeats in pof mutants. The observed distance is significantly smaller than the expected distance based on permutation analysis (38 bp vs. 132 bp, p<0.001). B. Histogram illustrating the distance between residual HP1a and repeats in egg mutants. The observed distance is significantly smaller than the expected distance based on permutation analysis (19 bp vs. 135 bp, p<0.001). (PDF) [file pgen.1002954.s015.pdf]

Figure S16

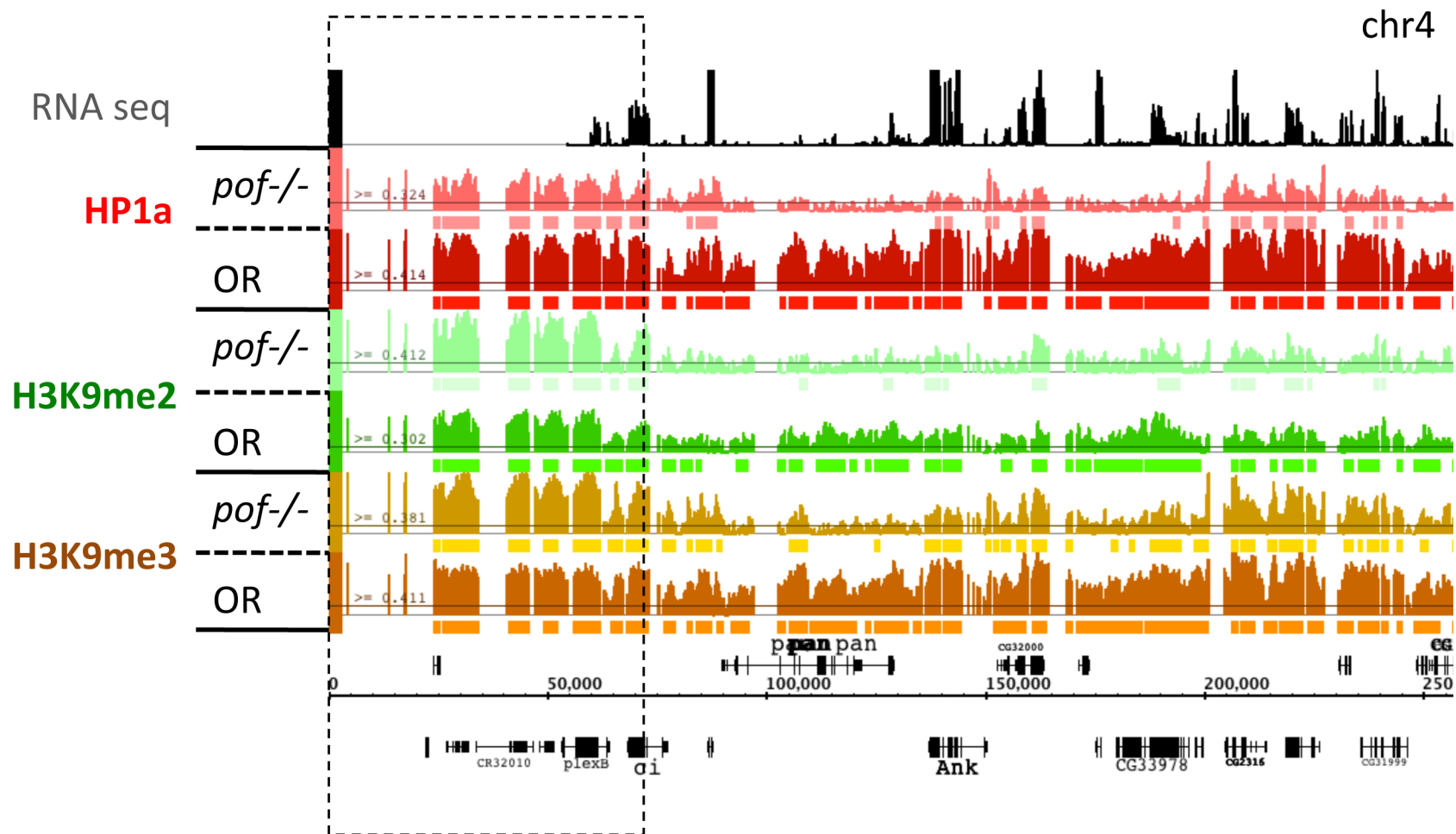

Supplement: Figure S16 — Heterochromatic marks are maintained at wildtype levels in the ∼70 kb of assembled sequence adjacent to the centromere in larvae lacking POF (pofD119). ChIP results from wildtype and mutant larvae are compared. The first panel shows RNA-seq data from wildtype larvae. X-axis: position along chromosome 4 in bp; Y-axis: ChIP enrichment for HP1a (top), H3K9me2 (middle), and H3K9me3 (bottom). (PDF) [file pgen.1002954.s016.pdf]
