# Supplementary material for: Enrichment of HP1a on Drosophila Chromosome 4 Genes Creates an Alternate Chromatin Structure Critical for Regulation in this Heterochromatic Domain
Source: PLoS Genet. 2012 Sep 20;8(9):e1002954. doi: 10.1371/journal.pgen.1002954 (PMC3447959; doi:10.1371/journal.pgen.1002954)
Supplement: Figure S5 — Chromosome 4 genes exhibit unique chromatin marks compared to genes in heterochromatin and euchromatin in S2 cells. Same analysis as shown in Figure S3, now with the same number of genes (N) as present on chromosome 4 randomly chosen from heterochromatin (Hetero) and euchromatin (Eu) as controls. (PDF) [file pgen.1002954.s005.pdf]

Figure S5

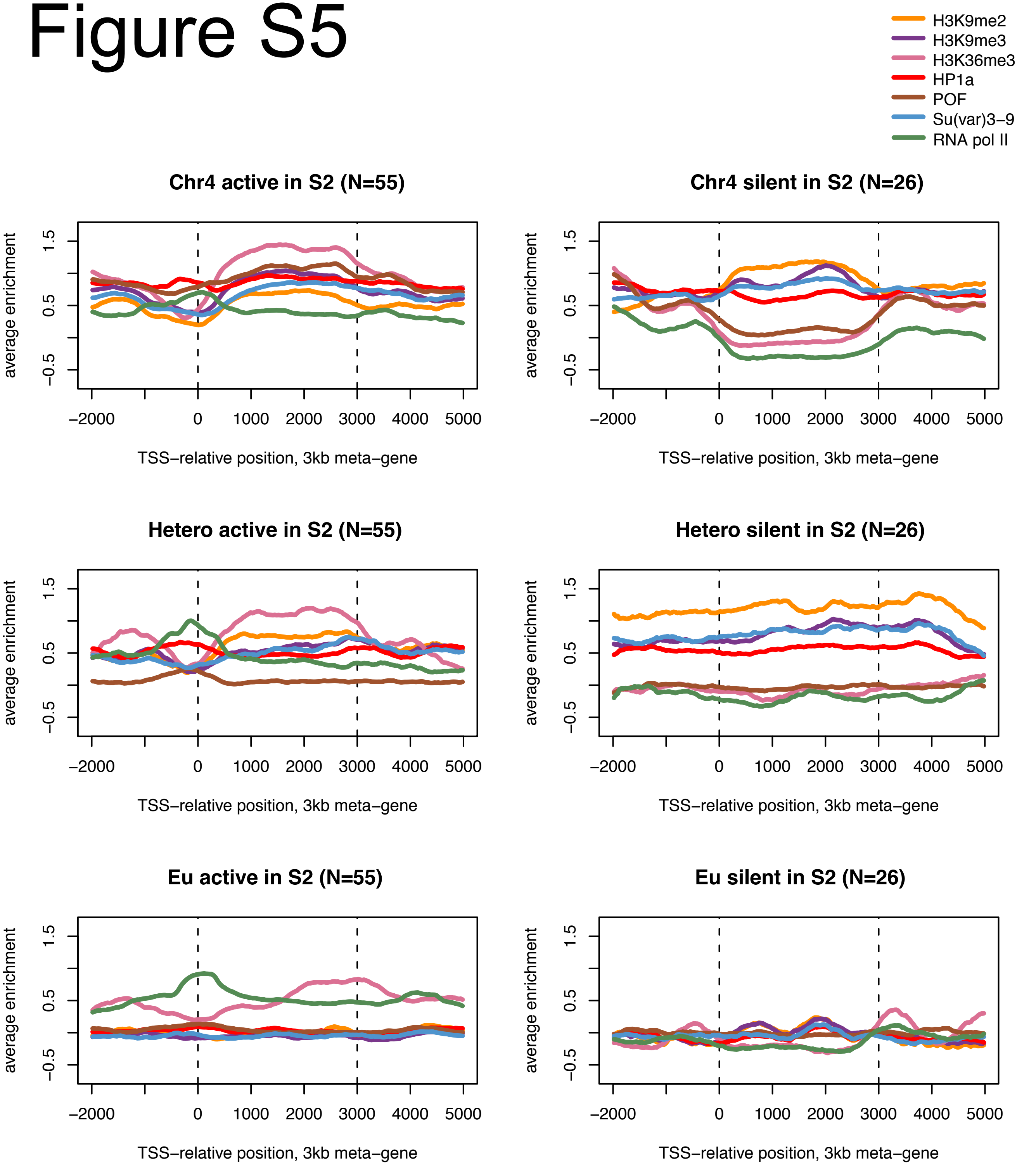
H3K9me2
H3K9me3
H3K36me3
HP1a
POF
Su(var)3−9
RNA pol II
Chr4 active in S2 (N=55)
Chr4 silent in S2 (N=26)
Hetero active in S2 (N=55)
Hetero silent in S2 (N=26)
Eu active in S2 (N=55)
Eu silent in S2 (N=26)
average enrichment
−0.5
0.5
1.5
−2000
0
1000
2000
3000
4000
5000
TSS−relative position, 3kb meta−gene
