# Supplementary material for: Enrichment of HP1a on Drosophila Chromosome 4 Genes Creates an Alternate Chromatin Structure Critical for Regulation in this Heterochromatic Domain
Source: PLoS Genet. 2012 Sep 20;8(9):e1002954. doi: 10.1371/journal.pgen.1002954 (PMC3447959; doi:10.1371/journal.pgen.1002954)
Supplement: Figure S7 — Histogram of the expected incidence of RNA pol II pausing on chromosome 4. Permutation analysis shows that the low pausing incidence on chromosome 4 is significantly different from that expected based on the pausing occurrence in euchromatin (A. p<3e-5) and pericentric heterochromatin (B. p<0.00024). (PDF) [file pgen.1002954.s007.pdf]

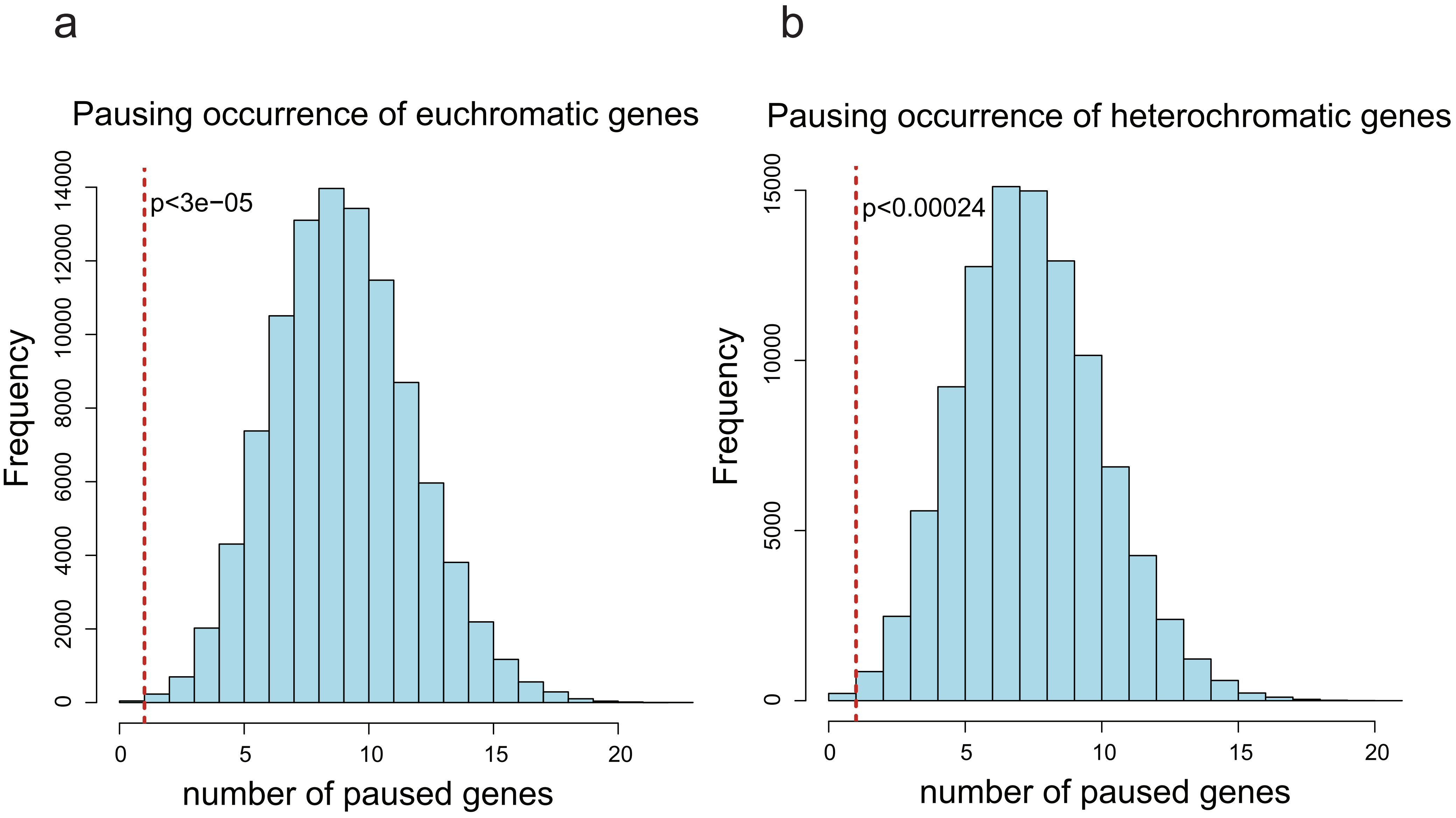
a
Pausing occurrence of euchromatic genes
Frequency
0
2000
4000
6000
8000
10000
12000
14000
p<3e−05
0
5
10
15
20
number of paused genes
b
Pausing occurrence of heterochromatic genes
Frequency
0
5000
10000
15000
p<0.00024
0
5
10
15
20
number of paused genes

Figure S7
