# Supplementary material for: Enrichment of HP1a on Drosophila Chromosome 4 Genes Creates an Alternate Chromatin Structure Critical for Regulation in this Heterochromatic Domain
Source: PLoS Genet. 2012 Sep 20;8(9):e1002954. doi: 10.1371/journal.pgen.1002954 (PMC3447959; doi:10.1371/journal.pgen.1002954)
Supplement: Figure S11 — Effect of HP1a depletion on RNA pol II pausing index. A. Ratio of the PI in mutants lacking HP1a compared to wildtype. The PI is defined as the ratio between the maximum enrichment value around TSS (+/−300 bp) and the medium enrichment values over the gene body (600 bp downstream of TSS to the end of the gene) [31]. 53 of 74 genes show an increase in PI, indicated by a ratio larger than 1. B. Histogram of RNA pol II level fold changes in HP1a mutants (log 2; average per gene) for genes on chromosomes 2, 3, and X (bottom panel) and genes on chromosome 4 (top panel), illustrating a decrease of RNA pol II levels for chromosome 4 genes. C. Relationship between RNA pol II level changes (Y-axis, in log 2) and expression level changes (X-axis, in log 10) of chromosome 4 genes in HP1a mutants compared to wildtype. Data points with x<0 and y<0 correspond to genes where both RNA pol II and expression levels decrease upon HP1a depletion (67 of 84 genes on chromosome 4; r = 0.4, Pearson correlation coefficient). (PDF) [file pgen.1002954.s011.pdf]

Figure S11

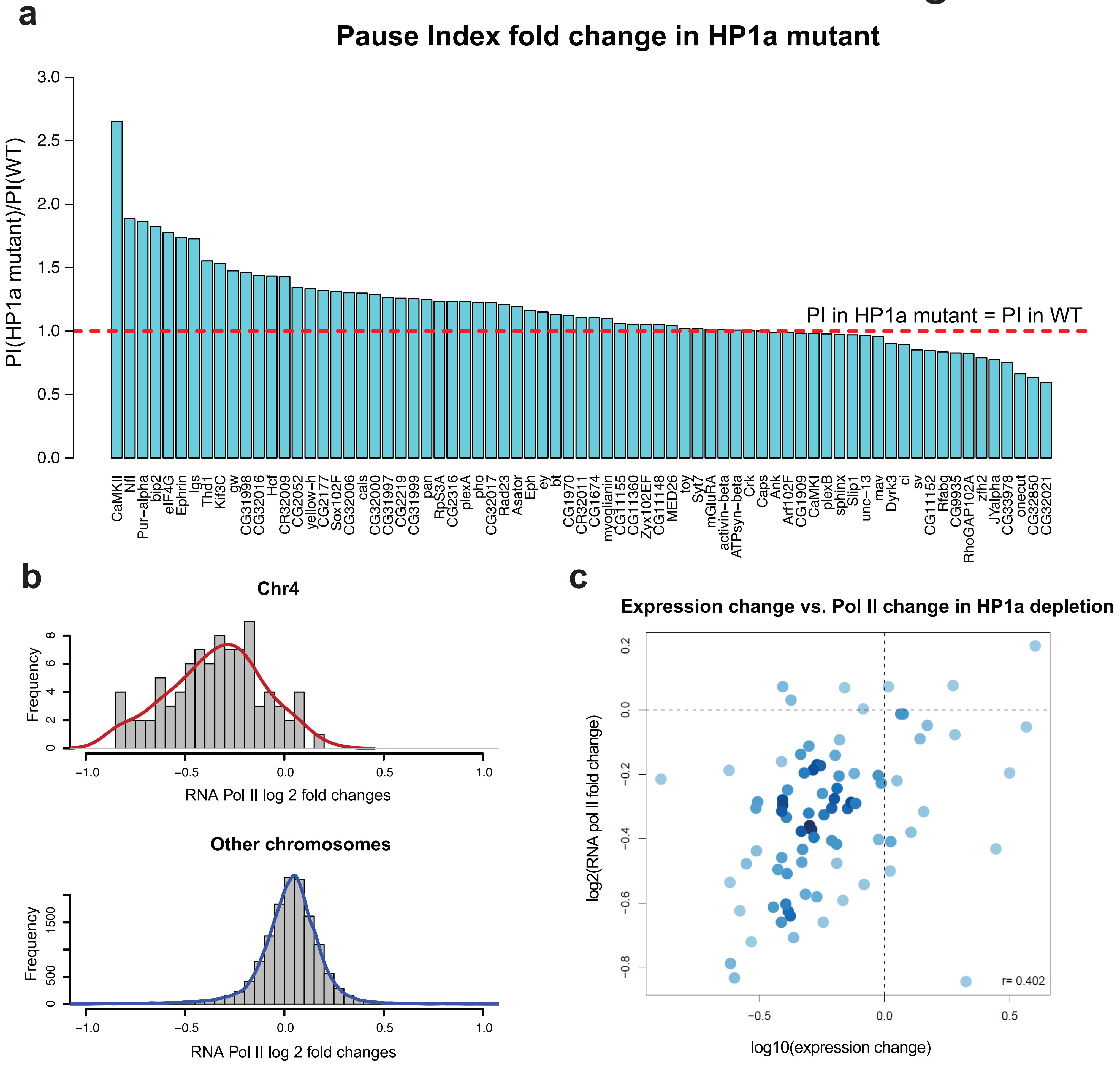

a
Pause Index fold change in HP1a mutant
PI(HP1a mutant)/PI(WT)
3.0
2.5
2.0
1.5
1.0
0.5
0.0
PI in HP1a mutant = PI in WT
CaMKII
Nfl
Pur−alpha
bip2
eIF4G
Ephrin
Igs
Thd1
Kif3C
gw
CG31998
CG32016
Hcf
CR32009
CG2052
yellow−h
CG2177
Sox102F
CG32006
cals
CG32000
CG31997
CG2219
CG31999
pan
RpS3A
CG2316
plexA
pho
CG32017
Rad23
Asator
Eph
ey
bt
CG1970
CR32011
CG1674
myoglianin
CG11155
CG11360
Zyx102EF
CG11148
MED26
toy
Syt7
mGluRA
activin−beta
ATPsyn−beta
Crk
Caps
Ank
Arf102F
CG1909
CaMKI
plexB
sphinx
Slip1
unc−13
mav
Dyrk3
ci
sv
CG11152
Rfabg
CG9935
RhoGAP102A
zfh2
JYalpha
CG33978
onecut
CG32850
CG32021
b
Chr4
Frequency
0
2
4
6
8
−1.0
−0.5
0.0
0.5
1.0
RNA Pol II log 2 fold changes
Other chromosomes
Frequency
0
500
1500
−1.0
−0.5
0.0
0.5
1.0
RNA Pol II log 2 fold changes
c
Expression change vs. Pol II change in HP1a depletion
log2(RNA pol II fold change)
0.2
0.0
−0.2
−0.4
−0.6
−0.8
−0.5
0.0
0.5
log10(expression change)
r= 0.402
