# Supplementary material for: Enrichment of HP1a on Drosophila Chromosome 4 Genes Creates an Alternate Chromatin Structure Critical for Regulation in this Heterochromatic Domain
Source: PLoS Genet. 2012 Sep 20;8(9):e1002954. doi: 10.1371/journal.pgen.1002954 (PMC3447959; doi:10.1371/journal.pgen.1002954)
Supplement: Table S3 — Polymerase pausing is rare on chromosome 4. Genes with paused RNA pol II have been identified by two methods. A. Polymerase pausing determined by GRO-seq analysis in S2 cells (Method 1). B. Genes with paused polymerase are identified solely based on ChIP-chip mapping of RNA pol II ([31]; Method 2). The percentage of paused genes is reported relative to the number of genes associated with RNA pol II. Note: In the table below, in A, the analysis is carried out on a per transcript basis. Thus, the total number of “genes” is inflated as some genes have multiple different transcripts and TSSs. (DOCX) [file pgen.1002954.s019.docx]

**Supplemental Table S3. Polymerase pausing is rare on chromosome 4.** Genes with paused RNA pol II have been identified by three methods. **A.** Polymerase pausing determined by GRO-seq analysis in S2 cells (Method 1). **B.** Genes with paused polymerase are identified solely based on ChIP-chip mapping of RNA pol II ([31]; Method 2). The percentage of paused genes is reported relative to the number of genes associated with RNA pol II.

Note: In the table below, in A, the analysis is carried out on a per transcript basis. Thus, the total number of “genes” is inflated as some genes have multiple different transcripts and TSSs.

**A.** S2 cells

|  | Euchromatin | Peric. heterochromatin | Chr. 4 |
| --- | --- | --- | --- |
| Total # of genes | 15886 | 624 | 116 |
| Genes with RNA pol II signal | 7556 (47.6%) | 155 (40.4%) | 63 (54.3%) |
| Paused genes | 1134 (15.0%) | 32 (12.5%) | 1 (1.6%)* |

* significantly lower than in euchromatin (p<0.00033)

**B.** S2 cells

|  | Euchromatin | Peric. heterochromatin | Chr. 4 |
| --- | --- | --- | --- |
| Total # of genes | 8722 | 457 | 79 |
| Genes with RNA pol II signal | 4003 (45.9%) | 147 (32.2%) | 49 (62.0%) |
| Paused genes | 590 (14.7%) | 24 (16.3%) | 3 (6.1%)* |

* significantly lower than in euchromatin (p<0.004)

BG3 cells

|  | Euchromatin | Peric. heterochromatin | Chr. 4 |
| --- | --- | --- | --- |
| Total # of genes | 8897 | 285 | 79 |
| Genes with RNA pol II signal | 3910 (43.9%) | 97 (34.0%) | 41 (51.9%) |
| Paused genes | 645 (16.5%) | 10 (10.3%) | 3 (7.3%)* |

* significantly lower than in euchromatin (p<0.00574)
