# Supplementary material for: Enrichment of HP1a on Drosophila Chromosome 4 Genes Creates an Alternate Chromatin Structure Critical for Regulation in this Heterochromatic Domain
Source: PLoS Genet. 2012 Sep 20;8(9):e1002954. doi: 10.1371/journal.pgen.1002954 (PMC3447959; doi:10.1371/journal.pgen.1002954)
Supplement: Table S4 — Developmental control genes are not depleted among chromosome 4 genes. GO term analysis demonstrates that the following terms are significantly enriched among chromosome 4 genes (p<0.05). No terms are significantly depleted. (DOCX) [file pgen.1002954.s020.docx]

**Supplemental Table S4. Developmental control genes are not depleted among chromosome 4 genes.** GO term analysis demonstrates that the following terms are significantly enriched among chromosome 4 genes (p<0.05). No terms are significantly depleted.

| GO ID | Term | # in reference | # in set | p value |
| --- | --- | --- | --- | --- |
| GO:0065007 | biological regulation | 2615 | 35 | 0.0013021 |
| GO:0030528 | transcription regulator activity | 685 | 16 | 0.0059633 |
| GO:0005488 | binding | 5698 | 56 | 0.0078702 |
| GO:0003700 | transcription factor activity | 402 | 12 | 0.0090615 |
| GO:0050794 | regulation of cellular process | 2227 | 30 | 0.0109882 |
| GO:0050789 | regulation of biological process | 2377 | 31 | 0.013914 |
| GO:0017154 | semaphorin receptor activity | 2 | 2 | 0.0380998 |
