# Supplementary material for: Enrichment of HP1a on Drosophila Chromosome 4 Genes Creates an Alternate Chromatin Structure Critical for Regulation in this Heterochromatic Domain
Source: PLoS Genet. 2012 Sep 20;8(9):e1002954. doi: 10.1371/journal.pgen.1002954 (PMC3447959; doi:10.1371/journal.pgen.1002954)
Supplement: Table S5 — The frequency of GAGA factor motifs, but not the pause button sequence, is underrepresented in chromosome 4 promoters. PB - Pause button. GAGA factor (TRL) binding motif – TRL motif. Inverted GAGA factor binding motif – iTRL motif. (DOCX) [file pgen.1002954.s021.docx]

**Supplemental Table S5. The frequency of GAGA factor motifs, but not the pause button sequence, is underrepresented in chromosome 4 promoters.** PB - Pause button. GAGA factor (TRL) binding motif – TRL motif. Inverted GAGA factor binding motif – iTRL motif.

|  | Euchromatin | Pericentric heterochromatin | Chromosome 4 |
| --- | --- | --- | --- |
| # of genes | 7556 | 256 | 63 |
| # of genes with PB | 1133 (15.0%) | 21 (8.2%)* | 6 (9.5%) |
| # of genes with TRL motif | 1832 (24.3%) | 62 (24.2%) | 7 (11.1%)** |
| # of genes with iTRL motif | 1902 (25.2%) | 70 (27.3%) | 8 (12.7%)*** |
| # of genes with Inr motif | 757 (10.0%) | 20 (7.8%) | 4 (6.4%) |

* significantly lower than euchromatin; p = 0.0038

** significantly lower than euchromatin (p < 2.69e-3) and heterochromatin (p = 2.67e-3)

*** significantly lower than euchromatin (p < 4.88e-3) and heterochromatin (p = 1.58e-3)
