# Supplementary material for: Enrichment of HP1a on Drosophila Chromosome 4 Genes Creates an Alternate Chromatin Structure Critical for Regulation in this Heterochromatic Domain
Source: PLoS Genet. 2012 Sep 20;8(9):e1002954. doi: 10.1371/journal.pgen.1002954 (PMC3447959; doi:10.1371/journal.pgen.1002954)
Supplement: Table S6 — The average Tm of 9-mer sequences downstream of TSSs on chromosome 4 is significantly lower than on other chromosomes. The minimum (min.), median, mean, and maximum (max.) melting temperature (Tm, °C) for 9-mer sequences in the first 100 bp downstream of all unique TSSs in the D. melanogaster genome are compared by chromosome arm. (DOCX) [file pgen.1002954.s022.docx]

**Supplemental Table S6. The average T_m_ of 9bp sequences downstream of TSSs on chromosome 4 is significantly lower than on other chromosomes.**

The minimum (min.), median, mean, and maximum (max.) melting temperature (T_m_, °C) for 9bp sequences in the first 100 bp downstream of all unique TSSs in the *Drosophila melanogaster* genome are compared by chromosome arm.

| **Chromosome** | **Min. T_m_** | **Median T_m_** | **Mean T_m_** | **Max. T_m_** |
| --- | --- | --- | --- | --- |
| **X** | 14.81 | 15.70 | 15.84 | 17.38 |
| **2L** | 14.15 | 14.98 | 15.03 | 16.23 |
| **2R** | 14.58 | 15.25 | 15.45 | 16.82 |
| **3L** | 14.04 | 14.96 | 15.09 | 16.41 |
| **3R** | 14.57 | 15.17 | 15.40 | 16.74 |
| **4** | 9.13 | 11.43 | 11.50 | 13.46 |
