# Supplementary material for: Enrichment of HP1a on Drosophila Chromosome 4 Genes Creates an Alternate Chromatin Structure Critical for Regulation in this Heterochromatic Domain
Source: PLoS Genet. 2012 Sep 20;8(9):e1002954. doi: 10.1371/journal.pgen.1002954 (PMC3447959; doi:10.1371/journal.pgen.1002954)
Supplement: Table S7 — HP1a and H3K9me2/3 enriched regions on chromosome 4 in wildtype, pofD119, Su(var)205, and egg10.1a mutant third instar larvae. Significantly enriched regions based on smoothed M-value profiles (FDR = 1e-3) are compared between wildtype and mutant. Note that the magnitude of the enriched peaks is not considered. POF+ region: POF enriched region in wildtype; POF- region: No POF enrichment in wildtype. HP1a+ region: HP1a enriched region in wildtype; HP1a- region: No HP1a enrichment in wildtype. (DOCX) [file pgen.1002954.s023.docx]

**Supplemental Table S7. HP1a and H3K9me2/3 enriched regions on chromosome 4 in wildtype, *pof^D119^*, *Su(var)205*, and *egg^10.1a^* mutant third larvae.** Significantly enriched regions based on smoothed M-value profiles (FDR=1e-3) are compared between wildtype and mutant. Note that the magnitude of the enriched peaks is not considered. POF+ region: POF enriched region in wildtype; POF- region: No POF enrichment in wildtype. HP1a+ region: HP1a enriched region in wildtype; HP1a- region: No HP1a enrichment in wildtype.

|  | **HP1a enriched**  **(bp)** | **HP1a (bp) in POF+ region** | **HP1a (bp) in POF- region** | **POF enriched (bp)** | **POF (bp) in HP1a+ region** | **POF (bp) in HP1a- region** |
| --- | --- | --- | --- | --- | --- | --- |
| **wildtype** | 668,454 | 347,199 | 321,255 | 351,879 | 347,199 | 4,689 |
| ***pof^D119^*** | 48,658  (7.3% of WT) | 16,347 | 32,311 | - | - | - |
| ***Su(var)205^04^/Su(var)205^05^*** | - | - | - | 281,581  (80% of WT) | 275,846 | 5,736 |
| ***egg^10.1a^*** | 112,219  (16.8% of WT) | 42,833 | 69,386 | 77,543  (18.0% of WT) | 77,543 | 0 |
|  |  |  |  |  |  |  |
|  |  |  |  |  |  |  |
|  | **H3K9me2 enriched (bp)** | **K9me2 (bp) in POF+ region** | **K9me2 (bp) in POF- region** | **H3K9me3 enriched (bp)** | **K9me3 (bp) in POF+ region** | **K9me3 (bp) in POF- region** |
| **wildtype** | 675,549 | 313.460 | 362,089 | 712,900 | 343,217 | 369,683 |
| ***pof^D119^*** | 267,638 (39.6% of WT) | 106,002 | 161,636 | 421,330 (59.1% of WT) | 220,128 | 201,202 |
| ***Su(var)205^04^/Su(var)205^05^*** | 74,797 (11.1% of WT) | 22,594 | 52,203 | 237,099 (33.3% of WT) | 163,908 | 73,191 |
| ***egg^10.1a^*** | 153,592 (22.7% of WT) | 60,752 | 92,840 | 138,288 (19.4% of WT) | 60,372 | 77,916 |
